# Macro-Meso-Micro Cross-Scale Correlation of Rheological Evolution in Waxy Crude Oil Emulsions under Dynamic Cooling Conditions

**DOI:** 10.1021/acsomega.6c08608

**Published:** 2026-09-14

**Authors:** Hang Dong, Jiajie Zhang, Sen Wang, Jian Zhao

**Affiliations:** Key Laboratory for Enhance Oil and Gas Recovery of the Ministry of Education, 117792 Northeast Petroleum University, Daqing 163318, Heilongjiang, China

## Abstract

The rheological deterioration of waxy crude oil emulsions poses a significant challenge to the flow assurance of subsea and long-distance pipelines. By integrating simultaneous rheology in situ microscopy with molecular dynamics (MD) simulations, the evolution of apparent viscosity was investigated under varying temperatures and shear rates. A cross-scale investigation was conducted to establish the correlations among macroscopic rheological response, mesoscopic structural evolution, and microscopic molecular interfacial behavior. The results indicate that the stepwise elevation in apparent viscosity during cooling is attributed to the formation of a composite network consisting of precipitated wax crystals and entrained water droplets. At a constant temperature (16 °C), the apparent viscosity exhibits a pronounced sensitivity to the water cut, increasing approximately 7.5-fold as the water cut rises from 10% to 40%. The mesostructure of high-water-cut systems is characterized by greater complexity, where dense droplets act as discrete resistance units that significantly impede fluid flow. Under shearing, the deconstruction of the three-dimensional wax-water network induces a distinct shear-thinning behavior. The heightened shear-thinning effect in high-water-cut emulsions is derived from the structural instability of Structure C composite units, which are dominated by heterogeneous interfaces and prone to disintegration. MD simulations further reveal a hierarchical mechanical resistance at the micro-interface, explaining the stability variations in mesoscopic aggregates. Specifically, the bonding strength of homo-aggregates significantly surpasses that of heterogeneous flocs interfaces; the latter exhibits interaction energy per unit area, peak stress, and fracture work of only 25.32%, 31.98%, and 10.96% of the former, respectively, identifying them as the structural weak points within the bulk mechanical network. Through the development of a quantitative model linking mesoscopic units with microscopic interfacial attributes, a meso-micro cross-scale correlation was established. The underlying mechanism is thus elucidated: macroscopic shear stress preferentially ruptures the heterogeneous bridging interfaces with lower micro-bonding energy, triggering a bottom-up cascading fragmentation of the mesoscopic network (Structure C → Structure B → Structure A), which macroscopically manifests as a sharp decline in apparent viscosity. Targeted weakening of the heterogeneous bridging interfaces provides an effective regulatory strategy for further enhancing the flowability of the system.

## Graphical abstract

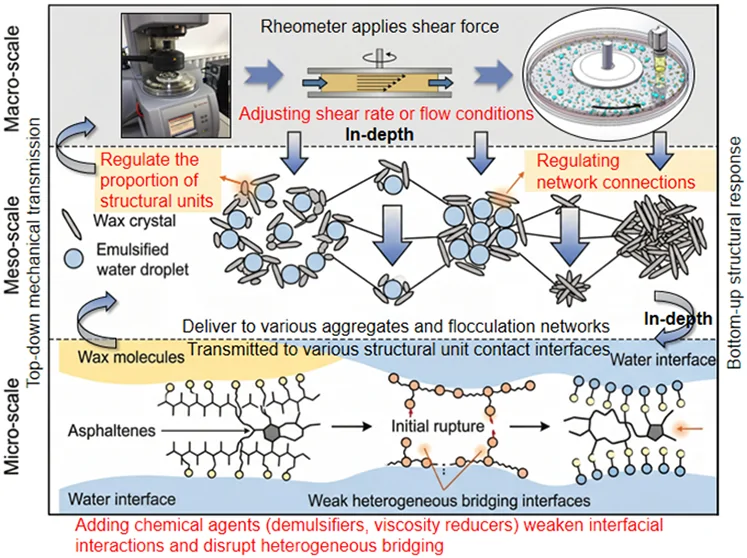

## 1 Introduction

Crude oil remains a paramount global energy resource. According to data from the International Energy Agency (IEA),^1^ petroleum accounts for over 30% of the global primary energy consumption, playing an indispensable role in the transportation and chemical sectors. As onshore oil and gas development matures, deep-water fields have emerged as a crucial frontier for reserve growth. In offshore operations, crude oil is typically transported via long-distance submarine pipelines to platforms or onshore processing facilities. In deep-sea environments, when the pipeline temperature drops below the wax appearance temperature (WAT), paraffin molecules precipitate and agglomerate into wax crystals. These crystals subsequently interlock to form a macroscopic three-dimensional network structure, which severely deteriorates the flowability of the crude oil and increases transportation resistance.^2^ Furthermore, during actual production and surface gathering operations, crude oil frequently exists as an emulsion. Due to the presence of natural surfactants such as asphaltenes and resins, the crude oil and produced water readily form water-in-oil (W/O) emulsions under the shear forces encountered in pipelines, valves, and separation equipment.^3^ Compared to single-phase waxy crude oil, the rheological behavior of waxy crude oil emulsions is significantly more complex. During the cooling process, pronounced interactions occur between the precipitating wax crystals and the emulsified water droplets. Specifically, the droplets alter the spatial distribution of the wax crystals, while the precipitated wax tends to accumulate at the oil-water interface. This mutual interaction forms wax-droplet composite structures, which dramatically elevate the apparent viscosity of the system and poses substantial challenges to pipeline flow assurance.

Extensive research has been conducted on the rheological properties and microscopic structures of waxy crude oil emulsions.^4–6^ Employing rheological measurements coupled with optical microscopy and Differential Scanning Calorimetry (DSC), Visintin et al.^7^ investigated the gelation behavior of waxy crude oils. They indicated that wax crystals interconnect via van der Waals forces to form a three-dimensional network, whose yield stress and moduli are significantly influenced by factors such as shear history and cooling rate. Through interfacial experiments, microscopic observations, and rheological testing, Chen et al.^8^ examined the stabilizing effect of wax crystals on W/O emulsions. The results indicate that wax crystals can adsorb onto the oil-water interface to form an elastic interfacial film, thereby participating in the construction of the macroscopic network. Ma et al.^9^ investigated the impact of wax crystal-water droplet aggregation on wax deposition under dynamic conditions, revealing that the composite aggregates formed by wax crystals and water droplets serve as critical microscopic units governing the deposition process. Furthermore, Dong et al.^10^ quantitatively analyzed the microstructural evolution of emulsions, elucidating the effects of temperature and shear rate on the aggregation behavior of water droplets and the apparent viscosity of the system. Additionally, Guo et al.^11^ studied the yield characteristics and thixotropy of emulsions with varying water cuts; Li et al.^12^ examined the influence of asphaltene concentration and temperature on wax-water droplet interactions; and Zhu et al.^13^ utilized molecular dynamics simulations to investigate the effects of cooling rate and asphaltene molecular structure on the aggregation of wax molecules. Recently, multi-scale correlation methods have been increasingly introduced into the rheological studies of complex fluids to bridge the gap between microscopic structures and macroscopic physical properties.^14^ However, existing research on waxy crude oil emulsions predominantly focuses on single-scale analyses or static conditions. There remains a notable lack of systematic, cross-scale synergistic mechanistic understanding. Specifically, under the combined effects of shear and cooling, the quantitative correlation between mesoscopic structural parameters and macroscopic apparent viscosity variations, as well as the impact of microscopic molecular interfacial behaviors on the disruption process of mesoscopic aggregates under shear, are yet to be thoroughly understood.

It should be noted that our group has previously investigated the dynamic behavior of wax crystals during the gelation of single-phase waxy crude oil using rheo-optic in situ measurement and CFD-DEM simulation.^15^ That work elucidated the mesoscale force-chain network evolution and its correlation with macroscopic viscosity. However, the system studied was a single-phase crude oil, and the simulation was at the particle scale. In contrast, the present study focuses on W/O waxy crude oil emulsions, which introduce a dispersed aqueous phase and thus form a more complex “wax crystal-water droplet-oil” multiphase system. More importantly, we employ molecular dynamics simulations to probe the interfacial interactions at the molecular scale, thereby extending the cross-scale correlation framework from the mesoscale (force chains) down to the microscale (molecular interfaces). The fundamental question addressed here is not how wax crystals form networks, but why heterogeneous interfaces dominate the shear-induced failure of emulsion networks.

Therefore, we propose a “macro-meso-micro” cross-scale correlation approach employing, synchronous rheo-microscopic experiments coupled with molecular dynamics (MD) simulations to investigate the gelation process and mechanical response mechanisms of waxy crude oil emulsions under dynamic cooling and shear conditions. Specifically, the investigated system is conceptualized across three distinct scales: (1) the macroscopic scale, represented by the rheological parameters of the bulk system (e.g., apparent viscosity); (2) the mesoscopic scale, characterized by the structural evolution and disruption processes of wax crystals, water droplets, and their resulting aggregates and flocs; and (3) the microscopic scale, focusing on intermolecular interactions and the configurational evolution of contact interfaces. The core mechanistic logic is that variations in the macroscopic apparent viscosity are fundamentally governed by the structural evolution and disruption of mesoscopic aggregates and flocs. Consequently, these mesoscopic behaviors intrinsically originate from the configurational evolution and rupture of molecular networks at the microscopic contact interfaces. The primary research contents are outlined as follows:

(1) Macro-meso synergistic law analysis: Using synchronous rheo-microscopic measurement, we obtain macroscopic viscosity data during dynamic cooling process under different water cuts (10%–40%) and shear rates (0.5 s^–1^, 2 s^–1^, 5 s^–1^), while synchronously capturing mesoscopic images of the spatial distribution and aggregation morphology of wax crystals and droplets. By extracting mesoscopic structural parameters (e.g., fractal dimension, minimum edge distance, and specific surface area), we establish a direct correlation between mesoscopic structural evolution and macroscopic apparent viscosity changes, thereby clarifying the mesoscopic physical basis for macroscopic rheological differences across water cuts.
(2) Meso-micro correlation mechanism exploration: We use molecular dynamics simulations were utilized to build a microscopic model containing wax and water molecules, and calculate the interaction energy changes on the microscopic interface. By applying stretching to the stabilized molecular aggregates, we analyze differences in adhesion strength and structural stability between homo-aggregate and hetero-aggregates contact interfaces. We then link the fracture characteristics of the microscopic interface were mapped to the destruction process of mesoscopic aggregates under shear action, and reveal the internal mechanisms of different aggregate structures showing different mechanical responses during shearing from the molecular level.
(3) Cross-scale mechanical behavior integration: We introduce the mechanical properties of microscopic interfaces (homo-aggregate, hetero-aggregate, hetero-floc contact interfaces) into the force analysis of mesoscopic units (Structures A, B and C). By analyzing the approximate proportion of each different microscopic interface in mesoscopic units, we explain the stability of mesoscopic structures in the emulsion network and clarify the root cause of the differences in macroscopic shear-thinning behavior. Finally, we close the cross-scale loop linking microscopic molecular configuration, mesoscopic structural evolution, and macroscopic rheological properties, thereby synergistically revealing the rheological mechanism of waxy crude oil emulsions across multiple scales.

## 2 Experimental and Simulation Methodology

### 2.1 Experimental Materials

The crude oil samples, sourced from the Hulunbuir oilfield, are classified as typical waxy crude oils.^15^ As the macroscopic rheological properties of such oils are highly sensitive to thermal and shear histories, a rigorous pretreatment protocol was implemented prior to the preparation of W/O emulsions. This procedure was designed to eliminate “history effects”, thereby establishing a consistent initial state across all samples and ensuring experimental reproducibility. The pretreatment involved heating the oil samples to 80 °C in a water bath, followed by a 2 h isothermal period under static conditions to ensure the complete dissolution of wax crystals and the complete dispersion of resin and asphaltene aggregates. Subsequently, the samples were cooled naturally to ambient temperature and subjected to static aging for a minimum of 48 h. This aging step established a uniform structural baseline across all test batches without altering the intrinsic chemical composition of the oil or the thermodynamic solubility of wax and asphaltenes. Immediately prior to emulsion preparation, the aged oil was reheated to 70 °C for 1 h—well above the wax disappearance temperature (WDT = 44.27 °C, determined via DSC)—to completely melt any crystalline wax structures formed during static aging and to fully redisperse asphaltene aggregates.^33,34^ The oil phase was subsequently equilibrated at 50 °C for 30 min before water addition. Since 50 °C is well above the measured wax appearance temperature (WAT = 39.46 °C), the continuous oil phase remained in a fully homogeneous, wax-free Newtonian state at the onset of emulsification. All W/O emulsions (10%, 20%, 30%, and 40% water cuts) were prepared using identical homogenization parameters (1000 rpm, 15 min, 50 °C). Consequently, the high-shear homogenization applied during emulsification acted solely on a liquid matrix free of crystalline wax, ensuring that the emulsification shear neither induced premature wax crystal nucleation nor altered the subsequent nucleation and growth dynamics of wax crystals during cooling.

The pretreated crude oil samples were characterized for their basic physicochemical properties. The wax appearance temperature (WAT) of the crude oil was determined using a differential scanning calorimeter (DSC, TA-Q2000; Figure 1) in accordance with the industry standard SY/T0545-2012. The results indicate that the WAT of the sample is 39.46 °C. By integrating the DSC thermogram (Figure 2) across the entire temperature range, the total enthalpy of wax crystallization was obtained, from which the wax content of the crude oil was calculated to be approximately 16.34 wt %. The compositional analysis of the crude oil was performed using the SARA (saturates, aromatics, resins, and asphaltenes) fractionation method via column chromatography. The analysis revealed that the crude oil comprises 65.8 wt % saturates, 18.0 wt % aromatics and naphthenes, 11.8 wt % resins, and 4.4 wt % asphaltenes. The density of the crude oil at 20 °C was measured to be 835.6 kg/m^3^ using an Anton Paar 4500M digital density meter, while its pour point was determined to be 21 °C according to the ASTM D5853 standard. Deionized water was utilized as the aqueous phase. To investigate the effect of water cut on the rheological behavior of the emulsions, W/O emulsions were prepared with varying water cuts of 10%, 20%, 30%, and 40% by volume. The carbon number distribution and detailed compositional properties of the crude oil are presented in Figure 3 and Table 1.

**Figure 1:**
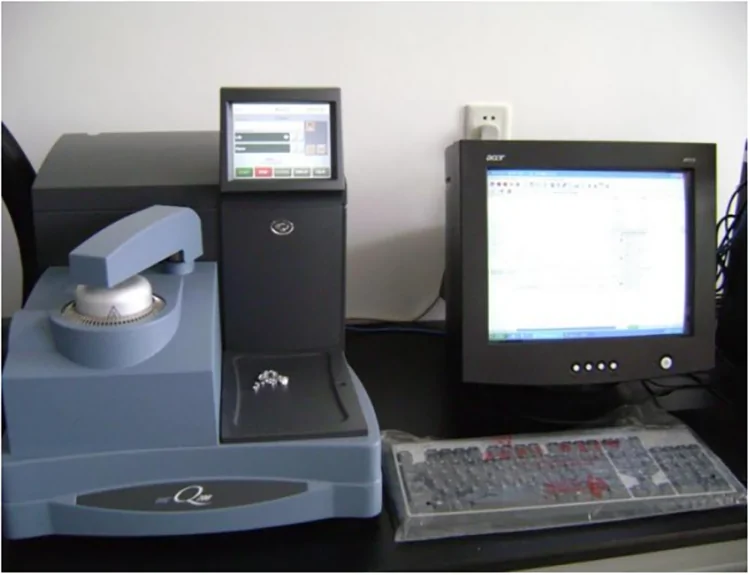
Differential scanning calorimeter (DSC).

**Figure 2:**
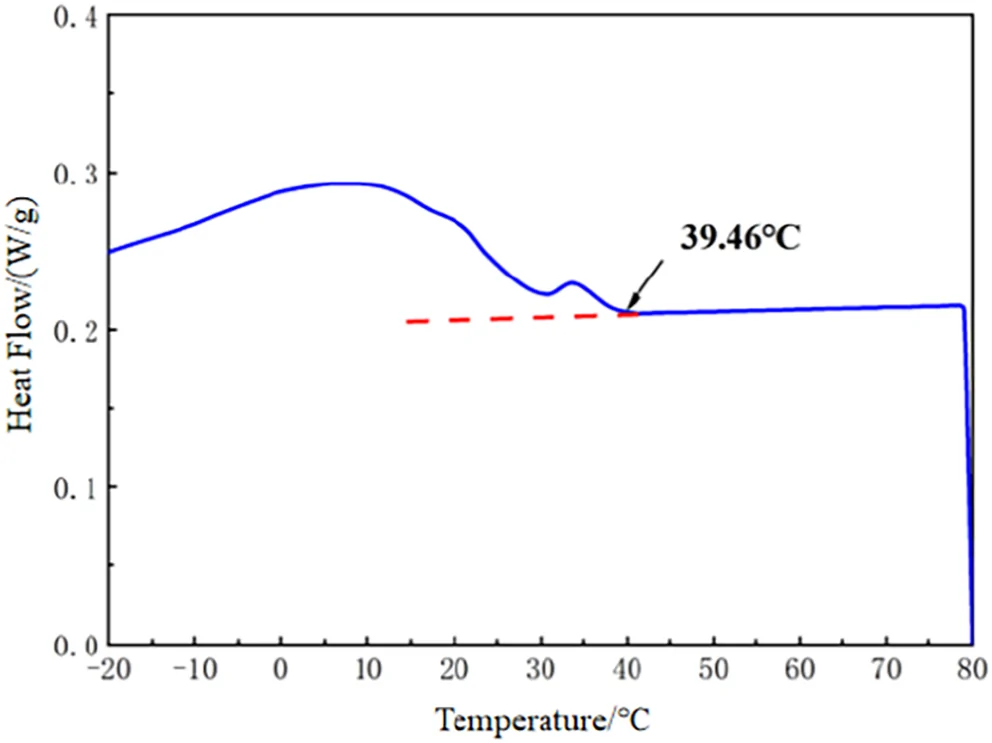
DSC thermogram of wax precipitation.

**Figure 3:**
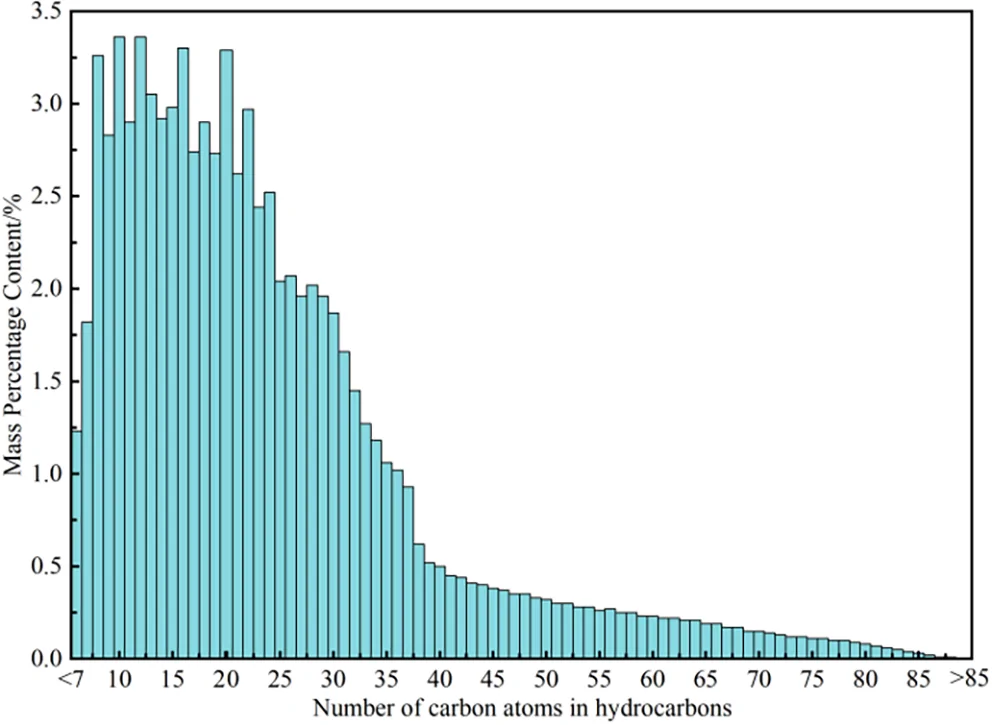
Carbon number distribution of the Hulunbuir crude oil.

**Table 1:** Mass Percentages of Different Carbon Number Ranges

Carbon number	<C20	C20–C37	C38–C77	C78–C89
Mass fraction (wt %)	39.38	34.33	10.8	0.56

### 2.2 Preparation of the Emulsions

To prevent wax precipitation during emulsification, the preparation temperature was set to 50 °C, which is well above the WAT of 39.46 °C. This ensures the continuous liquid state of the system throughout the process. Prior to emulsification, the pretreated crude oil samples were initially heated in an incubator at 70 °C to guarantee sufficient fluidity. Subsequently, they were transferred to a water bath at 50 °C and allowed to equilibrate thermally for a designated duration. The emulsions were formulated utilizing a single-step water addition approach. Specifically, a predetermined volume of deionized water was introduced into the crude oil under isothermal conditions, followed by homogenization using a high-speed variable-frequency homogenizer operating at 1000 rpm for 15 min. The selection of the optimal emulsification temperature necessitates a delicate balance between ensuring the complete dissolution of wax crystals and maintaining interfacial stability. Elevated temperatures may compromise the mechanical strength of the interfacial film, whereas excessively low temperatures could trigger premature wax precipitation. Based on preliminary experimental trials, a temperature of 50 °C was selected for emulsion preparation. At this temperature, stable emulsion systems were successfully obtained while effectively preventing the precipitation of wax crystals during the emulsification process.

### 2.3 Rheological Measurements

The dynamic cooling rheological measurements were conducted using an MCR-702 TwinDrive modular rotational rheometer (Anton Paar, Figure 4). A transparent quartz parallel-plate measuring system with a diameter of 43 mm was employed. This configuration created a transparent measuring gap between the upper and lower plates, thereby facilitating subsequent microscopic observations. Prior to the measurements, the rheometer stage was preheated to the designated initial temperature. Subsequently, a small volume of the emulsion sample was rapidly loaded onto the measuring area and allowed to thermally equilibrate under isothermal conditions for approximately 10 min. A standardized loading protocol was strictly followed for all water-cut samples, including consistent sample volume, plate-lowering speed, and edge-trimming procedure. This 10 min isothermal equilibration period served not only to achieve thermal equilibrium but also to fully relax any viscoelastic normal stresses or structural perturbations introduced by the brief shear encountered during sample loading, thereby ensuring a uniform initial shear history across all samples. Each water-cut condition was tested in at least three independent replicates, confirming excellent experimental reproducibility. To encompass the temperature range corresponding to the structural transition of the system from a liquid state to a gel-like network, the cooling process was initiated at the emulsion preparation temperature and terminated at 16 °C, with a constant cooling rate of 0.5 °C/min. During the dynamic cooling process, constant shear rates of 0.5, 2, and 5 s^–1^ were applied, respectively, while continuously recording the rheological curves of the apparent viscosity versus temperature.

**Figure 4:**
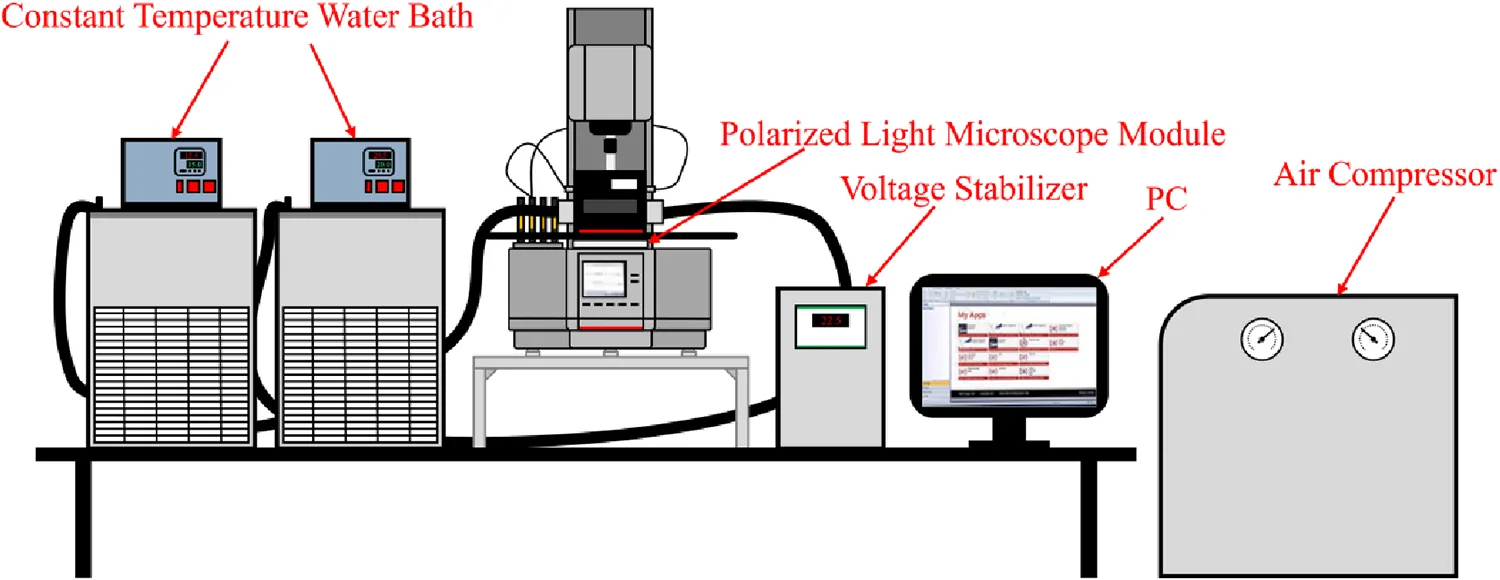
Anton Paar MCR-702 TwinDrive modular rotational rheometer.

### 2.4 In Situ Microscopic Observations

To monitor the structural evolution of the emulsions during shearing and cooling, a synchronous in situ rheo-microscopy system (Figure 5) was integrated with the aforementioned rheometer. Utilizing the transparent quartz parallel-plate geometry, the system enabled real-time optical imaging of the sample from below via a microscope. The imaging apparatus was equipped with a Lumenera CCD camera featuring a 2/3-in. color sensor, yielding an observation field of view of approximately 440 μm × 330 μm. The microscope observation window was fixed at a radial position corresponding to two-thirds of the plate radius (R_2/3_) from the center of the parallel-plate geometry. In the accompanying software of the Anton Paar MCR-702 rheometer, the CSR (Correction for Shear Rate) factor was set to R_2/3_, which enables the instrument to automatically match the local shear rate at the observation window position to the nominal shear rate configured in the software. Consequently, the shear rate values reported in the macroscopic viscosity curves are fully consistent with the actual local shear rate experienced at the microscopic observation point. Due to the inherently low light transmittance of waxy crude oils, the microscopic imaging quality was enhanced by integrating reflected and transmitted light sources, alongside the combined application of polarized and natural light. This, in conjunction with the built-in light source of the instrument, formed a multi-angle illumination configuration. Although the system inherently captures two-dimensional (2D) images, the dynamic kinematic behaviors and spatial rearrangements of both wax crystals and water droplets within the shear flow field could be effectively identified via continuous video recording and sequential image analysis. At elevated shear rates, the rapid movement of dispersed particles often induces motion blur during imaging. To circumvent this, a step-wise image acquisition strategy was implemented: upon reaching predetermined characteristic temperatures (40, 32, 24, and 16 °C), the shear rate was temporarily reduced to 0.5 s^–1^ for approximately 10 s. Once the optical capture was completed, the initial shear rate was promptly restored. This methodology successfully yielded high-clarity microscopic images at critical temperature nodes for analyzing the structural evolution of wax crystals and droplets, while strictly minimizing disruption to the thermal and shear histories of the emulsion system.^16^

**Figure 5:**
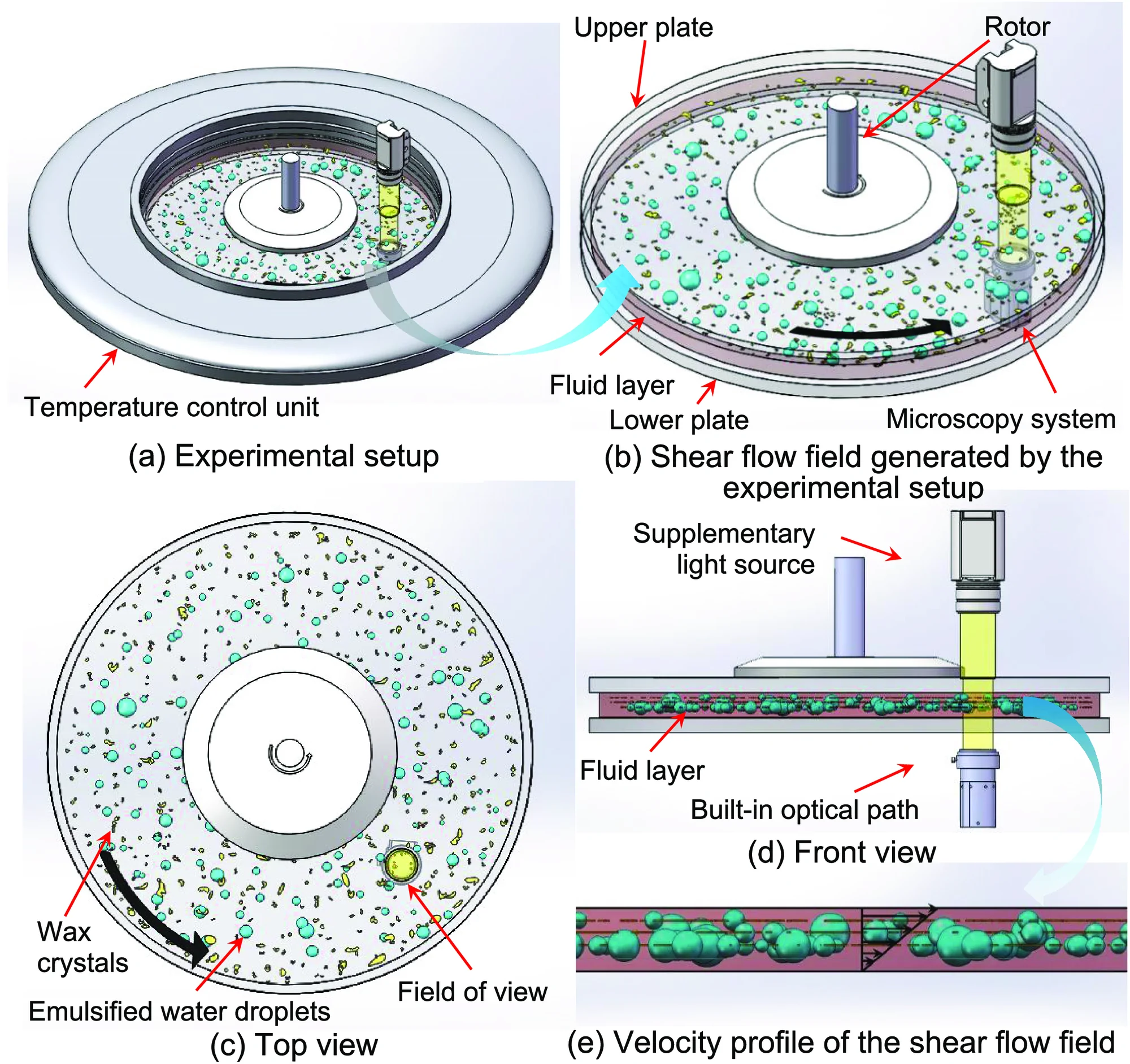
Schematic diagram of the synchronous in situ rheo-microscopy experimental setup.

### 2.5 Molecular Dynamics Simulations

#### 2.5.1 Construction of Simulation Models and Force Fields

The initial molecular configurations of each component were established. Geometric optimization of these structures was subsequently performed at the B3LYP/6-311G­(d,p) level of theory within the Density Functional Theory (DFT)^17^ framework using electronic structure software. To accurately characterize intermolecular electrostatic interactions, the electrostatic potentials were fitted using Multiwfn 3.8,^18,19^ and atomic partial charges were assigned via the Restrained Electrostatic Potential (RESP) method. Molecular topology files (.itp format) were generated utilizing the Sobtop^20^ tool based on the General Amber Force Field (GAFF), while water molecules were represented by the TIP3P model. The number of molecules of each component was determined based on the experimental data for carbon number distribution, density, and water cut. To simulate the wax phase, n-octadecane (C_18_H_38_) and n-octacosane (C_28_H_58_) were selected as representative short-chain and long-chain paraffin components, with 200 and 150 molecules, respectively. The aqueous phase comprised 2,300 water molecules, forming a nanoscale droplet model to represent the emulsified phase. The interfacial active fraction consisted of 40 molecules of asphaltenes (C_43_H_46_N_2_O_6_) and resins (C_24_H_32_S), with their molecular architectures referenced the established models of Boek et al.^21^ and Castellano et al.^22^ The molecular structures of resins and asphaltenes adopted in this study are widely recognized representative average-structure models in the literature, whose aromatic core sizes, heteroatom types, and overall compositions are consistent with typical waxy crude oils. The SARA analysis of the Hulunbuir crude oil (65.8 wt % saturates, 18.0 wt % aromatics, 11.8 wt % resins, 4.4 wt % asphaltenes) confirms that the selected models exhibit reasonable chemical compatibility with the experimental oil sample. It is acknowledged that real SARA fractions contain hundreds of distinct isomers and polyaromatic core structures, and that variations in heteroatom content (N, O, S) or island- versus archipelago-type architectures may quantitatively alter the computed interfacial energies. However, the present simulations are intended to compare the relative interfacial strengths of the three aggregate types rather than to reproduce the full chemical diversity of real crude oils, and this comparative hierarchy is governed by the chemical nature of each contact—compact van der Waals packing at homo-aggregate interfaces versus intrinsic polar–nonpolar incompatibility at hetero-aggregate interfaces—rather than by specific heteroatom decoration.^35–42^ A systematic parametric study of asphaltene molecular architecture effects is beyond the scope of this work and will be pursued in future investigations using customized molecular models based on the average molecular structure of specific crude oil samples. The corresponding molecular structures are illustrated in the following figure (Figure 6):

**Figure 6:**
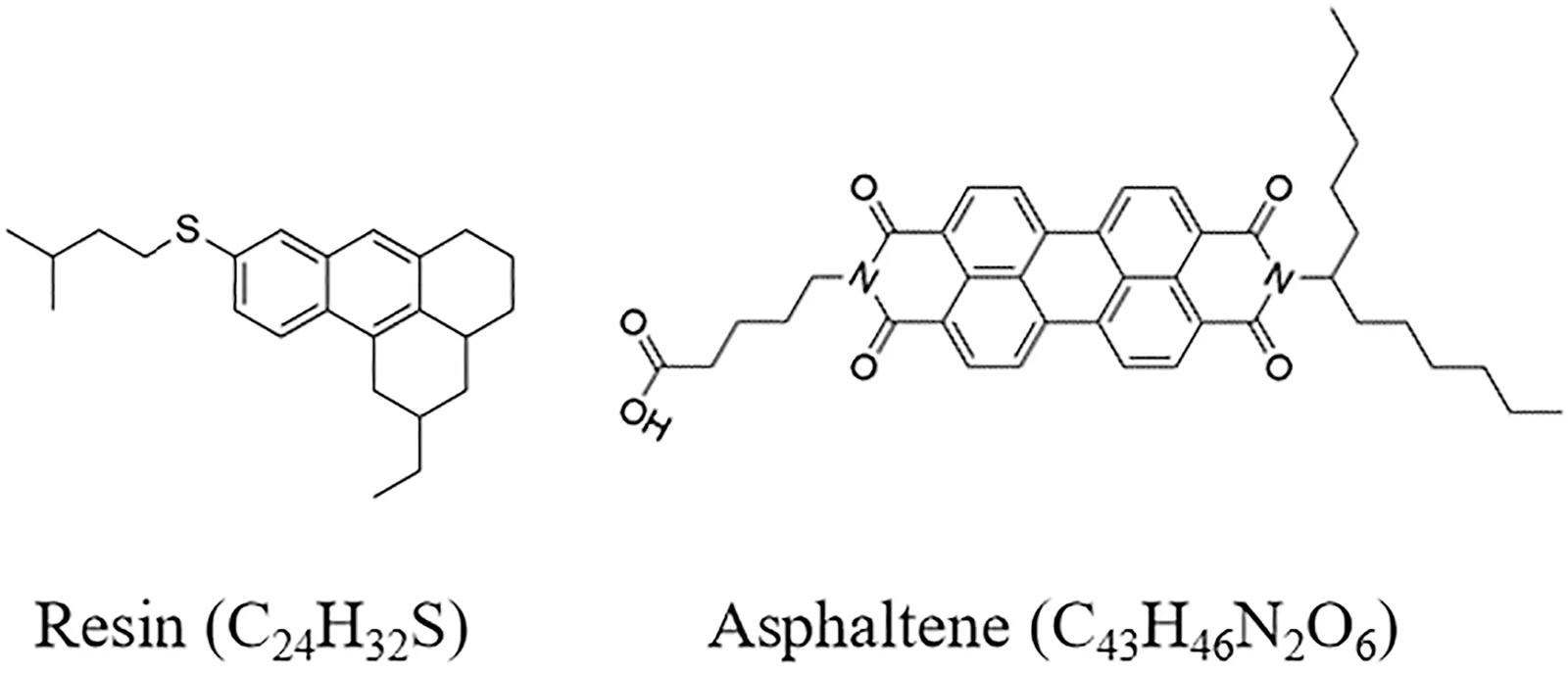
Molecular structures of the resin and asphaltene components.

The three aforementioned categories of components serve as relatively independent functional modules within the simulation. Their molar ratios are calibrated to match the experimental SARA analysis of the actual crude oil, ensuring compositional consistency between the simulation model and the real system. It should be highlighted that while droplets and wax crystals in actual waxy crude oil emulsions are typically at the micrometer scale, the direct construction of full-scale models via molecular dynamics (MD) simulations remains computationally prohibitive. Following established protocols in the literature, we constructed nanoscale water droplets and wax crystal clusters as simplified surrogates for their micrometer-scale counterparts to investigate the underlying interfacial interactions. Despite the inevitable disparity in length scales, the fundamental physical nature and the Structures of intermolecular forces (e.g., van der Waals forces and electrostatic interactions) between wax and water molecules remain invariant. Consequently, the interfacial interaction mechanisms and mechanical response characteristics derived from these nanoscale models provide meaningful insights into the behavior of the macroscale system. This multi-scale modeling strategy has been extensively validated and adopted in relevant research fields.^23,24^

#### 2.5.2 Initial System Configuration and Model Assembly

The molecular dynamics (MD) model for the contact interface was developed using a modular design strategy. This approach involves the preliminary construction and equilibration of representative component clusters within the emulsion system, followed by their controlled assembly at the interface. Initially, wax and water molecules were randomly distributed into primary simulation boxes of prescribed dimensions (5 × 5 × 5 nm^3^ for the wax crystal system and 8.5 × 8.5 × 8.5 nm^3^ for the water droplet system) utilizing the Packmol package.^25^ Subsequently, pre-formed wax crystal clusters and emulsified water droplet clusters were generated through a cooling-induced equilibration protocol.

During the interface assembly stage, diverse contact interface models were constructed using the aforementioned pre-formed clusters (as illustrated in Figure 7). For relatively straightforward interfacial interactions involving homo-aggregates (e.g., wax-wax and droplet–droplet) or hetero-aggregates (e.g., wax-droplet), a three-layer spatial configuration was employed. In this design, representative pre-formed clusters were positioned on both sides, while surfactant components (asphaltenes and resins) were concentrated in the central layer. A parallel spacing of 1.0 nm was maintained between adjacent layers, with the entire system enclosed in a 15 × 10 × 10 nm^3^ simulation box. For more complex heterogeneous flocculate interfaces involving multiple phase interactions (e.g., droplet-wax-droplet and wax-droplet-wax), a five-layer spatial configuration was implemented. In these models, the clusters and surfactant molecules were arranged in an alternating parallel sequence within a larger simulation box of 25 × 10 × 10 nm^3^. Based on these architectural principles, a total of 13 simulation cases were established. The detailed compositions and configuration parameters for each case are summarized in Table 2.

**Figure 7:**
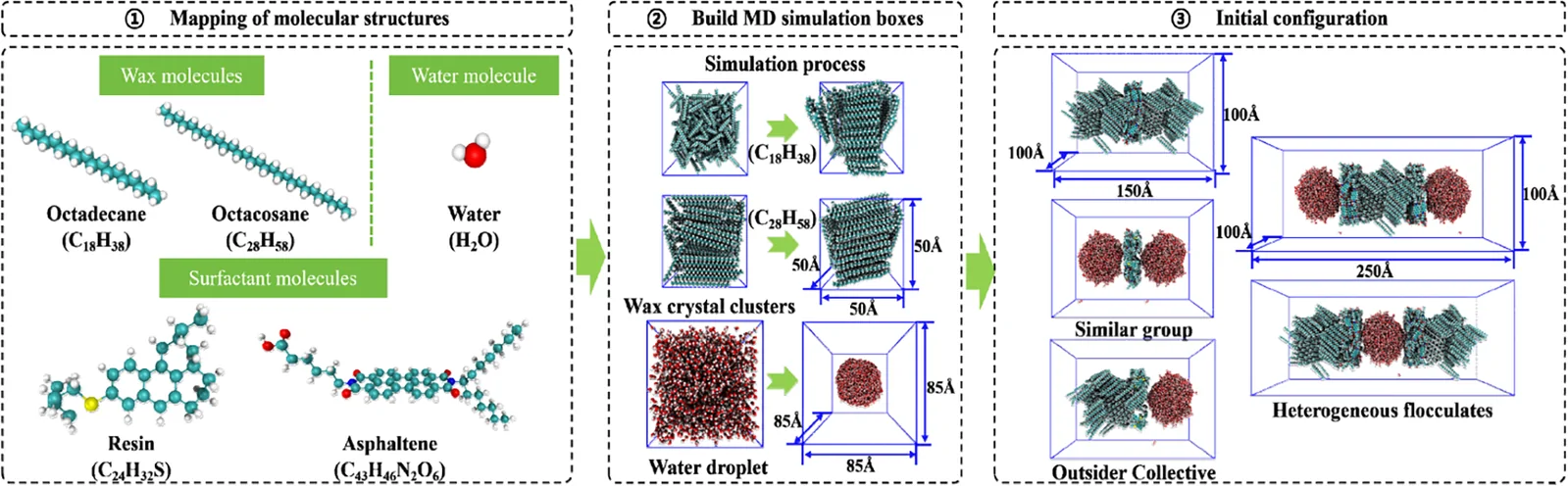
Flowchart of initial system generation and model assembly.

**Table 2:** Compositional Details and Configuration Parameters of the Simulation Models

Case	Molecular Contact Model	C_18_H_38_	C_28_H_58_	C_43_H_46_N_2_O_6_	C_24_H_32_S	H_2_O	Initial box size (nm^3^)	Stretching box size (nm^3^)
1	Wax-Wax	400	0	40	0	0	15 × 10 × 10	100 × 10 × 10
2	Wax-Wax	400	0	20	20	0	15 × 10 × 10	100 × 10 × 10
3	Wax-Wax	0	300	40	0	0	15 × 10 × 10	100 × 10 × 10
4	Wax-Wax	0	300	20	20	0	15 × 10 × 10	100 × 10 × 10
5	Wax-Wax	200	150	20	20	0	15 × 10 × 10	100 × 10 × 10
6	Droplet–Droplet	0	0	20	20	4600	15 × 10 × 10	100 × 10 × 10
7	Wax-Droplet	200	0	0	40	2300	15 × 10 × 10	100 × 10 × 10
8	Wax-Droplet	200	0	40	0	2300	15 × 10 × 10	100 × 10 × 10
9	Wax-Droplet	200	0	20	20	2300	15 × 10 × 10	100 × 10 × 10
10	Droplet-Wax-Droplet	200	0	80	0	4600	25 × 10 × 10	200 × 10 × 10
11	Droplet-Wax-Droplet	200	0	40	40	4600	25 × 10 × 10	200 × 10 × 10
12	Wax-Droplet-Wax	400	0	80	0	2300	25 × 10 × 10	200 × 10 × 10
13	Wax-Droplet-Wax	400	0	40	40	2300	25 × 10 × 10	200 × 10 × 10

Upon equilibration of the interfacial contact systems, the largest aggregate cluster—defined by a molecular distance cutoff of 0.35 nm—was extracted for subsequent tensile simulations. For the simple contact systems constructed with the three-layer design, the simulation box was extended to 100 × 10 × 10 nm^3^; for the complex five-layer contact systems, it was extended to 200 × 10 × 10 nm^3^. This extension was implemented to ensure sufficient spatial buffers, thereby preventing unphysical artifacts associated with periodic boundary conditions as the cluster undergoes elongation.

#### 2.5.3 Simulation Details and Protocols

Molecular dynamics (MD) simulations were conducted using the GROMACS 2022.5 package,^26^ with periodic boundary conditions (PBC) applied in all three dimensions. Energy minimization was performed via the steepest descent algorithm until the maximum force converged below 10.0 kJ·mol^–1^·nm^–1^. All simulations were executed in the NVT ensemble with a time step of 2 fs. The LINCS algorithm was employed to constrain all hydrogen-containing bonds, ensuring numerical stability. Temperature control was managed by the V-rescale thermostat, while long-range electrostatic interactions were computed using the Particle Mesh Ewald (PME) method. For the nucleation stage, initial atomic velocities were assigned according to the Maxwell-Boltzmann distribution at 100 K. The total simulation spanned 10 ns, comprising a 2 ns linear heating phase from 100 to 303.15 K, followed by an 8 ns isothermal equilibration. The aggregation phase involved a pre-equilibration step (annealing from 100 to 313.15 K over 100 ps), followed by a 100 ns production run: a linear cooling from 313.15 to 299.15 K over 20 ns and a subsequent 80 ns isothermal run to facilitate cluster formation. The center-of-mass (COM) motion was removed every 100 steps using the “Linear” mode to prevent global drift. In the tensile simulations, the umbrella pulling algorithm was employed to investigate the fracture process, using a wax crystal-wax crystal system as a representative model. The largest wax crystal cluster within the system was bisected based on its spatial midline oriented along the tensile direction. This partition yielded two distinct regions: a reference fixed group (Wax_A) and a tensile group (Wax_B), representing the stationary and displaced components of the cluster, respectively. The pulling velocity was set at 0.001 nm/ps with a harmonic spring constant of 1000 kJ·mol^–1^·nm^–2^. This pulling velocity corresponds to a linear separation rate of approximately 1 m/s.. It should be emphasized that the simulations apply a normal-separation (tensile) mode, whereas the macroscopic rheological experiments impose simple shear; the two deformation modes are governed by distinct physical mechanisms, so a direct conversion into an equivalent macroscopic shear rate is not strictly rigorous. For a rough scale comparison, approximating the macroscopic parallel-plate gap as 1 mm yields an apparent shear rate on the order of 10^3^ s^–1^, which is roughly two to three orders of magnitude higher than the experimental shear rates of 0.5–5 s^–1^. At such elevated strain rates, interfacial molecules have insufficient time for full conformational relaxation, leading to strain-hardening effects and consequently to overestimated peak stresses and works of fracture compared with quasi-static conditions.^43–46^ This rate effect is expected to be a systematic bias that acts uniformly on all three interfacial categories, and the relative ranking of interfacial strengths is therefore expected to remain unchanged, because it is governed by intrinsic chemical and packing differences rather than by the pulling rate.^45,46^ Therefore, although the absolute magnitudes of peak stress and work of fracture reported here are rate-sensitive and should be interpreted with caution, the relative interfacial strengths and the mechanistic insights derived from the simulations remain valid for interpreting the experimental observations. The value of 0.001 nm/ps was selected as a compromise between computational feasibility and the need to fully capture the interfacial rupture process, and it falls within the range commonly adopted in steered molecular dynamics studies of interfacial separation.^44^ These runs were also conducted under NVT conditions, with force–displacement data recorded every 1000 steps for subsequent mechanical response analysis.

#### 2.5.4 Post-Processing and Statistical Analysis

The post-processing of simulation trajectories and the visualization of microstructural evolution were primarily conducted using the GROMACS built-in analysis suite and VMD software.^27^ To evaluate the interaction strength of various contact interfaces, the inter-group distances, non-bonded interaction energies (comprising van der Waals and electrostatic components), and other parameters were extracted across different simulation stages. Two configuration-oriented strategies were implemented for energy quantification based on the specific physical processes:

(1) Initial Configuration-Oriented Approach: This method is primarily employed to characterize the adsorption and aggregation phases. In this framework, discrete clusters in their initial states are defined as the reference index groups. This allows for the precise quantification of potential energy evolution as the system transitions from a dispersed state to a consolidated aggregated state.
(2) Result Configuration-Oriented Method (Reverse Tracing): Specifically applied to the analysis of tensile fracture simulations. Index groups are defined based on the two distinct fragments completely separated at the simulation terminus. By tracing these groups back to the onset of the stretching phase, the energy evolution trajectory is extracted. This strategy effectively eliminates interference from non-ideal fractures caused by molecular entanglement, ensuring that energy calculations strictly reflect the real physical separation at the interface.

Following the extraction of dynamic force profiles and energy-time sequences, data integration and visualization were performed using professional scientific plotting software. It should be noted that the nanoscale droplets and wax clusters employed in the MD simulations are orders of magnitude smaller than the experimental droplets and wax crystals, and this scale disparity warrants careful discussion. First, regarding line tension: at the nanoscale, the ratio of the three-phase contact line length to the interfacial area scales inversely with aggregate size, such that line tension (typically 10^–11^–10^–10^ N^47,48,50^ ) contributes more significantly to the total interaction energy than in macroscopic systems. Nevertheless, since all interaction energies reported here are normalized by the effective contact area, this boundary contribution is substantially diluted upon area averaging; moreover, it acts as a correction common to all three aggregate types and thus does not affect their relative ranking. Second, regarding interfacial curvature: curvature corrections to interfacial properties are quantified by the Tolman length, which is of molecular dimension,^49^ and molecular simulations of nanodroplets have shown that interfacial properties converge to the planar-interface limit for droplet radii beyond a few nanometres.^51,52^ In any case, the interfacial hierarchy identified here is governed by chemical compatibility and molecular packing efficiency—compact van der Waals stacking of homologous hydrocarbon chains at homo-aggregate interfaces versus inherently weak polar–nonpolar interactions at hetero-aggregate interfaces—which are intrinsic to the molecular species and therefore scale-invariant. Third, regarding spatial confinement: because non-bonded interactions are short-ranged, local interfacial packing and per-unit-area interaction energies are governed by correlations extending over only a few molecular diameters, and per-unit-area non-bonded interaction energies have been shown to converge toward macroscopic values for aggregate sizes beyond a few nanometres.^52,53^ Therefore, the absolute magnitudes of the reported mechanical parameters should be interpreted with caution, whereas the relative ranking of interfacial strengths and the qualitative mechanistic insights derived from the MD simulations remain valid for interpreting the experimental observations.

### 2.6 Data Processing and Statistical Analysis

To quantitatively interpret the rheological data and microscopic observations, specialized image processing algorithms were employed to perform grayscale conversion and threshold segmentation on CCD-captured color micrographs, enabling the identification of morphological contours for both droplets and wax crystal particles. Subsequently, geometric parameters of the structural units within the field of view were extracted for statistical characterization. Specifically, the spatial uniformity of the dispersed phase within the shear flow field was evaluated via the specific surface area, while the inter-particle minimum edge distance was measured to distinguish between dispersed and aggregated states. Furthermore, the perimeter fractal dimension was introduced to quantitatively describe the irregular boundary features of the composite structures under shear. Simultaneously, the kinematic characteristics of the particles were estimated through coordinate tracking across consecutive frames to determine displacement patterns. These microscopic insights were further correlated with the previously discussed molecular-scale interaction energies and interfacial evolution features to provide a multi-scale validation of the underlying mechanisms.

## 3 Experimental and MD Simulation Results and Discussion

### 3.1 Macro-Mesoscopic Rheological Performance of W/O Waxy Crude Oil Emulsions during Cooling

The rheological properties of waxy crude oils exhibit a pronounced sensitivity to temperature fluctuations. The transition in rheological behavior during cooling is fundamentally governed by thermodynamically driven phase transition processes. As the temperature declines, paraffin molecules precipitate as solids, transforming the oil from a single-phase Newtonian fluid into a complex liquid-solid non-Newtonian system with increasingly intricate rheological characteristics.^28^ In waxy crude oil emulsions, the interactions between water droplets and wax crystals further complicate the rheological response compared to pure crude oils.^29^ Beyond the influences of water cut and temperature, the system’s behavior is significantly dictated by the applied shear rate. To investigate the effects of water cut and shear rate on the gelation characteristics of W/O waxy crude oil emulsions, a synchronous rheo-microscopy technique was employed. This study systematically explored the viscosity-temperature characteristics of Hulunbuir waxy crude oil emulsions with varying water cuts (10%, 20%, 30%, and 40%) under dynamic cooling conditions. The experiments were conducted at shear rates of 0.5, 2, and 5 s^–1^, with a terminal temperature of 16°C and a constant cooling rate of 0.5 °C/min. The synchronous rheological and microscopic observations obtained across different water cuts are presented below:

As illustrated in Figure 8, at a constant water cut, the viscosity of the waxy crude oil emulsions increases progressively with declining temperature, exhibiting distinct staged growth characteristics. Under isothermal conditions, the system viscosity rises proportionally with increasing water cut. By correlating the synchronous rheo-microscopic observations from Figure 9 with the evolution of microstructural parameters extracted in Figure 10, the viscosity-temperature curves can be categorized into three distinct stages for detailed analysis.

**Figure 8:**
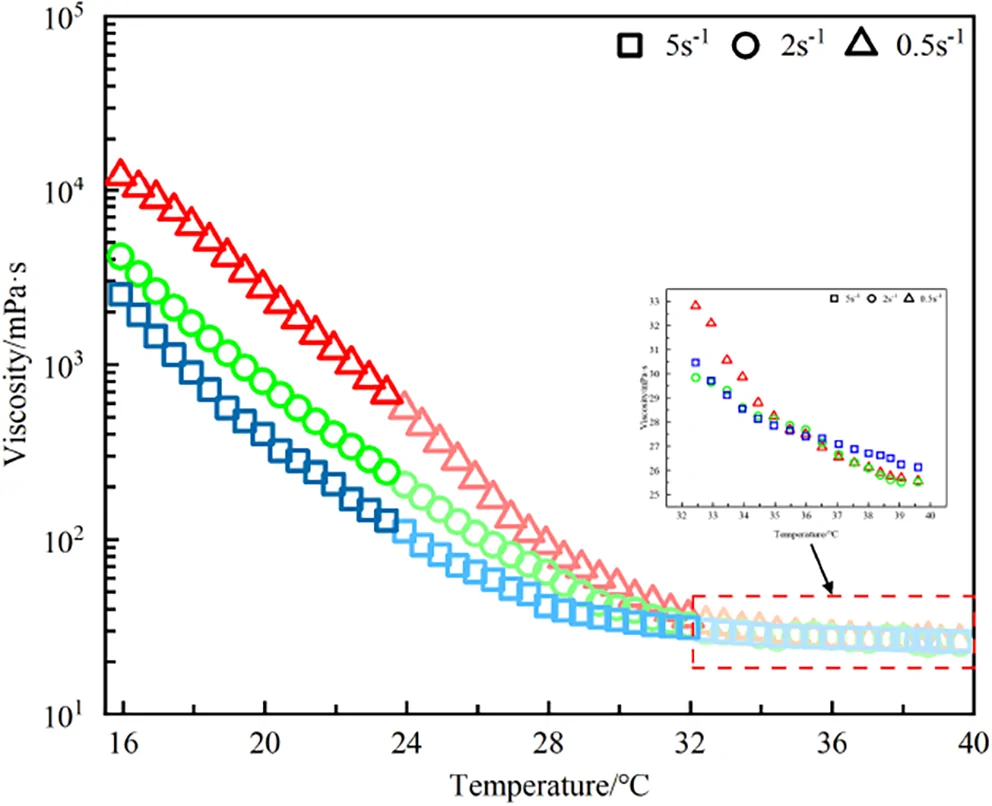
Synchronous rheo-microscopic characterization of waxy crude oil emulsions with varying water cuts (Rheological Results).

**Figure 9:**
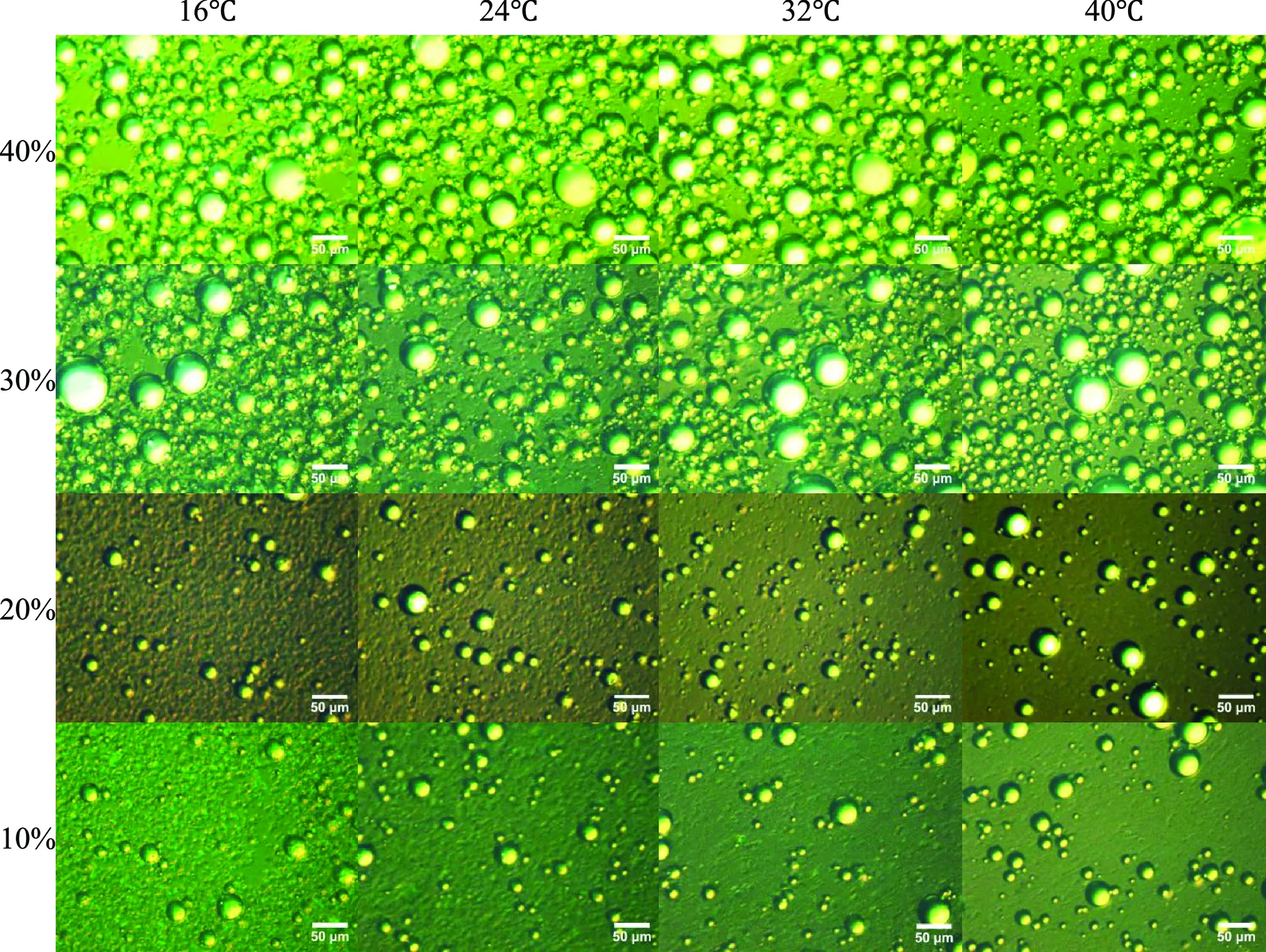
Synchronous rheo-microscopic characterization of waxy crude oil emulsions with varying water cuts (Microscopic Results).

**Figure 10:**
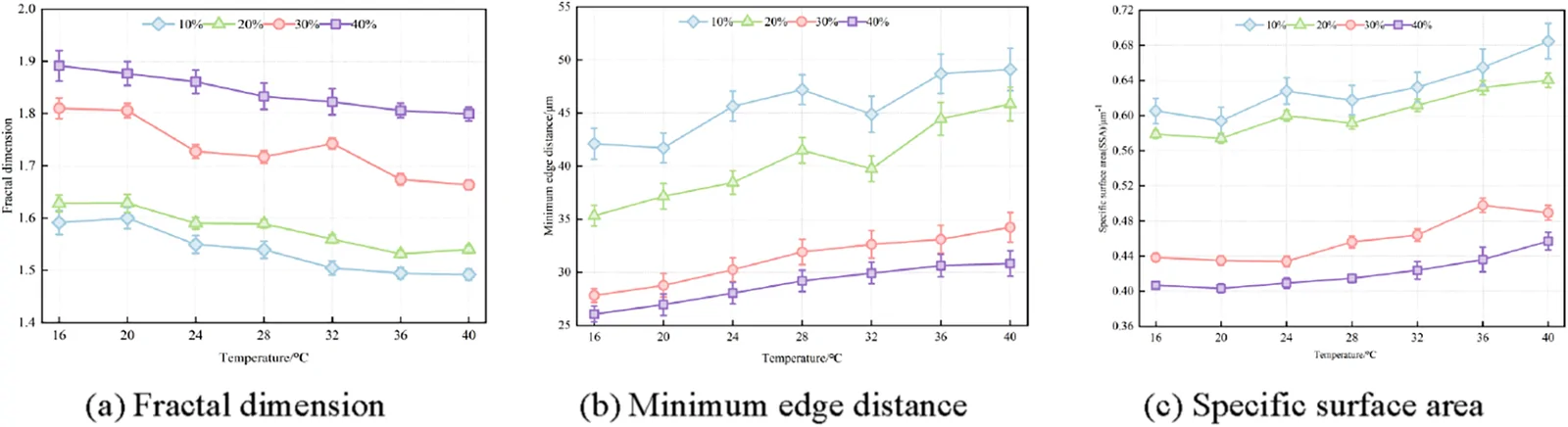
Evolution of mesoscopic quantitative parameters during the cooling process.

Stage I (40 °C–32 °C): This phase corresponds to the initial precipitation of wax crystals, characterized by a marginal increase in bulk viscosity. Microscopic observations reveal that at a lower water cut (10%), wax crystals and water droplets predominantly exist as dispersed individual entities at higher temperatures. Their spatial distribution remains uniform, and the limited quantity of precipitated wax crystals is insufficient to formulate a continuous flocculated network, thereby exerting minimal restriction on the flow field. Consequently, the apparent viscosity peaks at merely 25.559 mPa·s during this stage. To verify the above judgment based on microscopic observation, mesoscopic structural quantitative parameters are introduced for characterization. The data indicates a consistently low fractal dimension within this temperature regime (averaging 1.497). Concurrently, the minimum edge distance between structural units remains substantial (averaging 47.566 μm), accompanied by a relatively high specific surface area (averaging 0.657 μm^–1^). These metrics collectively suggest infrequent inter-particle contacts and a loose, monomer-dominated structural morphology. This trend is in excellent agreement with the microscopic observations, where droplets and wax crystals are observed to migrate relatively unimpeded within the flow field and readily detach upon collision. Conversely, as the water cut escalates to 40%, the abundance and dimensions of the dispersed droplets surge significantly. This elevated dispersed-phase volume fraction amplifies the hydrodynamic resistance against the flow field, diminishing the system’s mobility under shear. Consequently, the isothermal viscosity exhibits a drastic surge from 25.559 mPa·s to 397.88 mPa·s. It is noteworthy, however, that within high water-cut systems, the fluctuations in structural parameters remain marginal across this specific temperature window. The fractal dimension registers a negligible increment from 1.799 to 1.822, and the minimum edge distance persists at a high level (declining marginally from 30.822 μm to 29.918 μm). This underscores the absence of pronounced wax crystal aggregates. Therefore, the pronounced viscosity enhancement in this stage is fundamentally attributable to the augmented hydrodynamic resistance induced by the increased dispersed-phase volume, rather than the development of a structural wax network.

Stage II (32 °C–24 °C): As the temperature drops further, the system exhibits an accelerated increase in viscosity. Micrographs reveal an enhanced precipitation and growth rate of wax crystals. Driven by intermolecular forces, wax particles progressively collide and coalesce to form wax aggregates. Compared to Stage I, the mesoscopic structural parameters across all water cuts undergo distinct variations: the fractal dimension of the wax-droplet aggregates increases, whereas the minimum edge distance and specific surface area decrease. These quantitative shifts signify an elevated collision probability among dispersed particles, driving the structural transition from isolated entities to complex aggregates and augmenting mesoscopic heterogeneity. At lower water cuts, wax-wax aggregation dominates, initiating the development of a rudimentary wax network. Conversely, as the water cut rises, a fraction of the wax aggregates begins to co-flocculate with the water droplets, yielding wax-droplet co-aggregates. At elevated water cuts (e.g., 40%), the spatial gap between droplets narrows significantly, allowing wax crystals to act as bridging agents. This leads to the formation of intricate droplet-wax-droplet composite structures. This microstructural evolution is corroborated by the parameter variations: higher water cuts yield a greater fractal dimension (increasing from 1.822 to 1.861), alongside a reduced minimum edge distance (from 29.918 μm to 28.037 μm) and specific surface area (from 0.424 μm^–1^ to 0.409 μm^–1^), indicating a highly compacted structural configuration. Consequently, the isothermal viscosity at 24 °C surges drastically from 165.24 mPa·s (at a 10% water cut) to 2807.6 mPa·s (at a 40% water cut). Therefore, the rapid viscosity enhancement during this phase is primarily governed by the formation of composite aggregates, driven by the synergistic interactions between wax networks and emulsified droplets.

Stage III (24 °C–16 °C): As the cooling progresses, the system viscosity experiences a further and substantial escalation. With the continuous decline in temperature, wax crystals persistently precipitate, grow, and aggregate, eventually forming a dense flocculated network. Under isothermal conditions, the system viscosity escalates significantly with an increasing water cut, surging from 3504.81 mPa·s (at a 10% water cut) to 26,156 mPa·s (at a 40% water cut). Microscopic observations reveal that at a lower water cut (10%), the structural framework is predominantly composed of wax aggregates or flocs. The water droplets are embedded within this matrix, encapsulating liquid hydrocarbons inside the flocculated architecture. As the water cut rises (to 20% and 30%), inter-droplet aggregation emerges. A fraction of these droplets substitutes the wax crystals within the original wax network, yielding a droplet-wax composite flocculated structure. When the water cut further escalates to 40%, an extensive quantity of droplets and wax crystals closely inter-stack, trapping liquid hydrocarbons and forming a highly intricate multiphase flocculated network. Correspondingly, the variations in mesoscopic structural parameters become increasingly pronounced. The fractal dimension continues its upward trajectory to a maximum of approximately 1.891. Concurrently, the minimum edge distance further contracts from 28.037 μm to 26.059 μm, and the specific surface area drops slightly from 0.409 μm^–1^ to 0.406 μm^–1^. These quantitative shifts indicate highly compacted inter-particle linkages, confirming the densification of the structural network. Although elevated water cuts result in the partial replacement of wax-wax linkages by droplets, the resulting composite droplet-wax architecture significantly amplifies the overall structural complexity. This heightened complexity drastically intensifies the hydrodynamic resistance, ultimately manifesting as an abrupt surge in the macroscopic viscosity.

The integration of rheological testing and micrographs analysis reveals that the system viscosity increases with the water cut fraction. This phenomenon is attributed not only to the increment in the dispersed phase volume fraction but also to the evolution of the mesoscale architecture. From a hydrodynamic perspective, an increase in water cut leads to a higher droplet population density. Microscopic statistical data indicate that the average droplet count per field of view rises from 109 at 10% water cut to 150, 501, and 552 at 20%, 30%, and 40% water cut, respectively, accompanied by larger droplet sizes and higher degrees of aggregation. During shear flow, these droplets act as discrete flow-resistance units within the streamlines. The proliferation, enlargement, and subsequent aggregation of droplets enhance the impedance to fluid trajectories, thereby resulting in a higher apparent viscosity under equivalent shear rates. Conversely, at low water cut, the sparse droplet distribution exerts minimal resistance to the flow field, maintaining a lower bulk viscosity. From a mesostructural standpoint, increasing water cut significantly alters the spatial organization of wax crystals and water droplets. The increased droplet count facilitates bridging and attachment of wax crystals between droplets, fostering the formation of wax-droplet aggregates or complex droplet-wax-droplet structures. These composite structures are substantially larger than individual dispersed phase particles. In waxy crude oil emulsions, while typical emulsified water droplets range from 10 to 100 μm and individual wax crystals vary between 20 and 85 μm, the characteristic dimensions of the captured co-aggregates can reach several multiples of a single droplet’s diameter. Specifically, droplets encapsulated within wax deposits can exhibit diameters up to 115 μm, with the overall aggregate size expanding further upon peripheral wax crystal attachment. As the water cut increases, these co-aggregates interconnect to form large-scale flocs that occupy spatial domains far exceeding those of individual components. The resulting enhanced spatial coverage, coupled with increased structural complexity and three-dimensional connectivity, leads to the development of a sophisticated flocculation network. This is further evidenced by the increased fractal dimension and decreased inter-unit spacing and specific surface area, which directly contribute to the observed rise in macroscopic viscosity. It should be pointed out that although quantitative analysis of microstructure can explain the phenomenon that increased water cut leads to structural complication and thus causes viscosity increase at the mesoscale, it is still difficult to fundamentally clarify why increased water cut promotes the formation of more complex aggregate structures of wax crystals and droplets. This issue involves wax crystal surface components, interfacial interactions, and molecular-scale interaction mechanisms between different components. Consequently, it is essential to employ molecular dynamics (MD) simulations from a microscopic perspective to investigate the wax crystal surface structure and interfacial interaction energies. Such analysis aims to reveal the underlying mechanisms of water cut on wax-droplet interactions and structural formation, which will be further explored in subsequent simulation studies.

### 3.2 Macro-Mesoscopic Manifestations of the Effect of Shear Rate on the Rheological Properties of W/O Waxy Crude Oil Emulsions

Waxy crude oil is a typical non-Newtonian fluid that exhibits pronounced shear-thinning behavior under applied shear; that is, the apparent viscosity of the system decreases as the shear rate increases. This behavior originates from the orientation, disruption, and rearrangement of the internal structural network under the influence of the shear flow field (Figure 13). As illustrated in Figures 11 and 12, at a constant water cut, the apparent viscosity of the W/O waxy crude oil emulsion decreases as the shear rate increases, exhibiting a shear-thinning behavior analogous to that of pure waxy crude oil. This indicates that under the influence of the shear flow field, the spatial structural network within the emulsion—co-assembled by wax crystal aggregates, emulsified water droplets, and surface-active components—undergoes gradual rearrangement and continuous disruption. At low shear rates, the wax crystals precipitated during cooling establish a robust three-dimensional (3D) structural skeleton, driven by van der Waals forces and the adsorption of polar components such as resins and asphaltenes. Concurrently, the dispersed emulsified water droplets embed into this matrix, creating localized composite structural units with the surrounding wax crystals and interfacial active components. This complex network effectively hinders the mobility of the continuous oil phase, thereby yielding a high apparent viscosity. As the shear rate escalates, the intensified shear forces progressively dismantle the inter-crystal interactions. Simultaneously, the flow field induces the deformation, migration, and redistribution of the emulsified droplets, weakening the linkages between the structural units. Consequently, the wax aggregates disintegrate and align parallel to the flow direction. The interfacial linkages involving water droplets are highly susceptible to slippage and rearrangement, leading to the continuous unraveling of the original spatial network. This reduction in internal structural resistance enhances the fluidity of the continuous oil phase, ultimately causing the macroscopic apparent viscosity to decline. Furthermore, comparative analysis reveals that emulsions with varying water cuts exhibit distinct sensitivities to shear. To quantitatively characterize these differential structural responses, a novel parameter termed “shear-thinning intensity” is introduced. This parameter delineates the relative magnitude of apparent viscosity reduction across the applied shear rate range. Calculated based on the apparent viscosities at the initial and terminal shear rates, the shear-thinning intensity for systems with varying water cuts is comparatively illustrated in a bar chart (the formula is presented below, and the results are shown in Figure 14)­

1 $$I_{\mathrm{st}}\left(w\right)=\frac{\eta_{1}-\eta_{2}}{\eta_{1}}\times 100\%$$

Where *I*_*st*_ *(w)* represents the shear-thinning intensity at a specific water cut *w*(%), η_*i*_ is the apparent viscosity at the initial shear rate (Pa·s), η_*f*_ denotes the apparent viscosity at the terminal shear rate (Pa·s), with γ_2_ > γ_1_.

**Figure 11:**
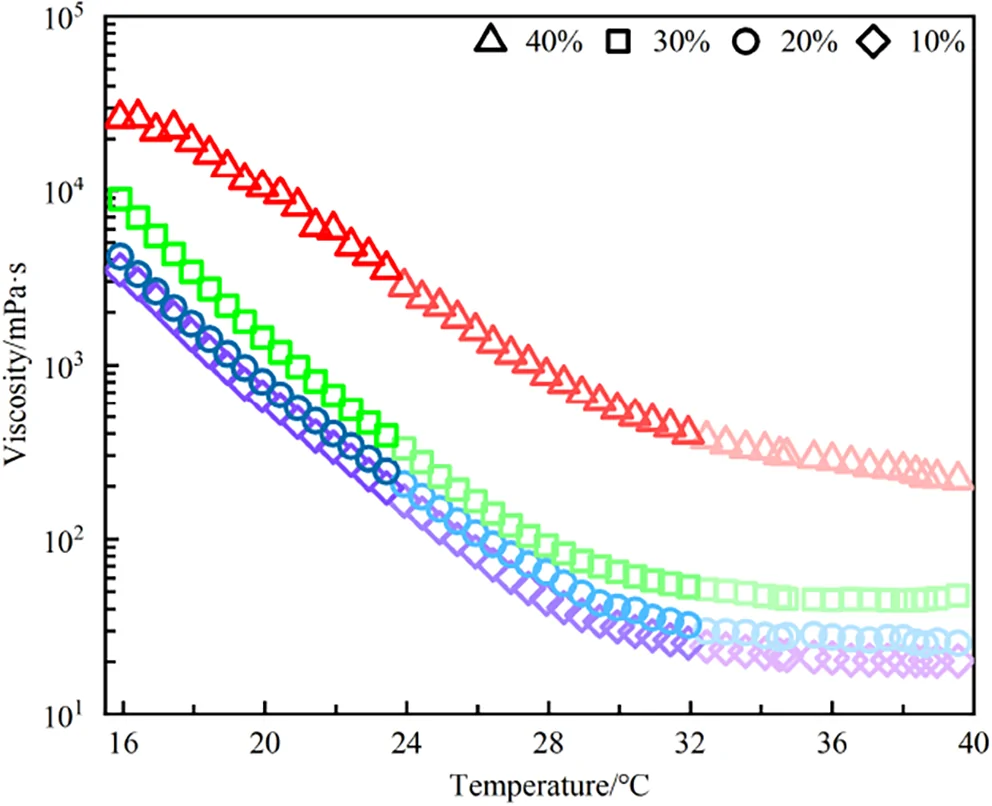
Rheological-in-situ microscopic synchronous test results of waxy crude oil emulsions under different shear rates (Rheological results).

**Figure 12:**
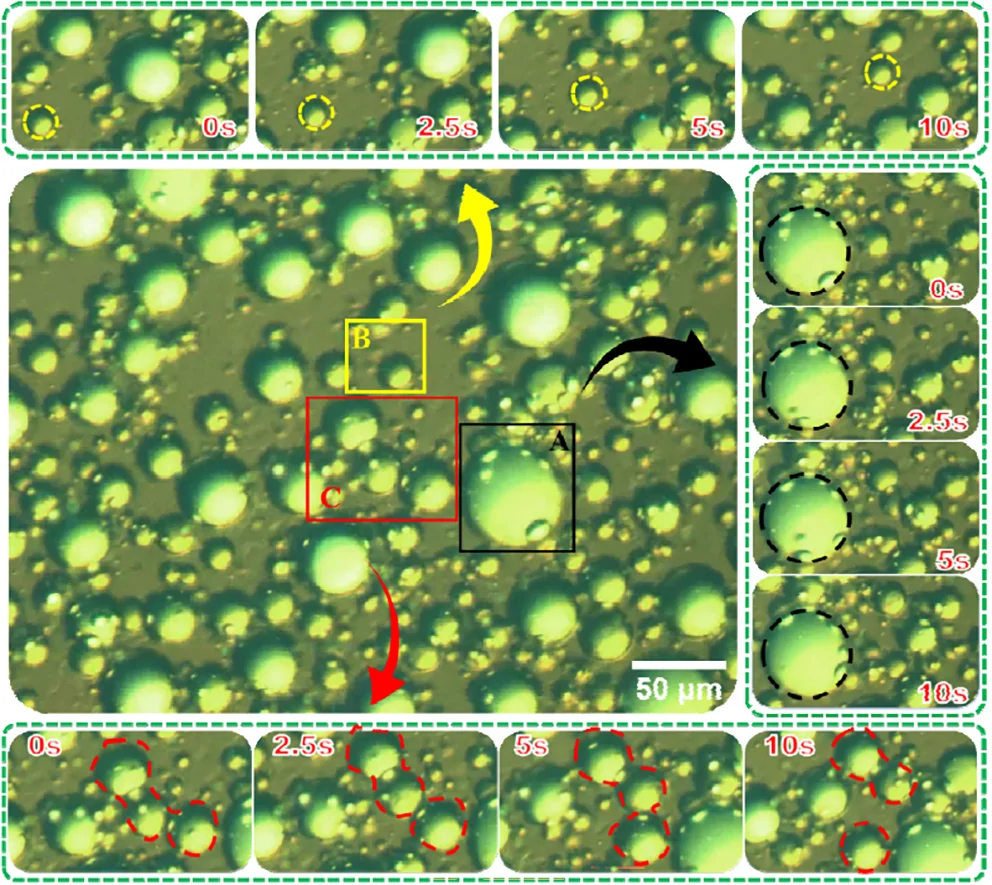
Mesoscopic structures A, B, and C in waxy crude oil emulsions at different moments (Images extracted from video).

**Figure 13:**
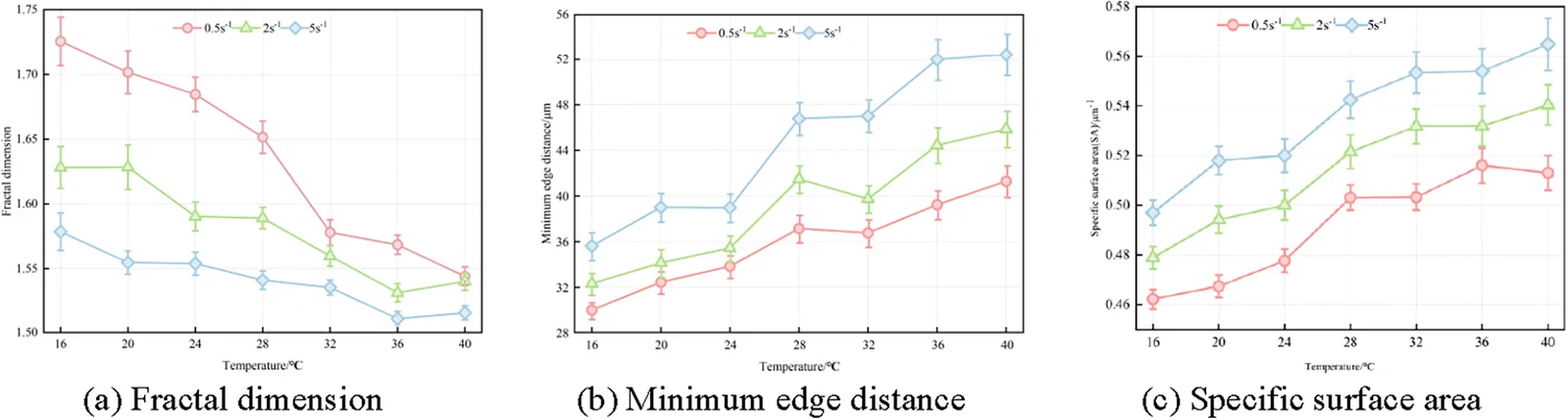
Evolution of mesoscopic quantitative parameters during the cooling process.

**Figure 14:**
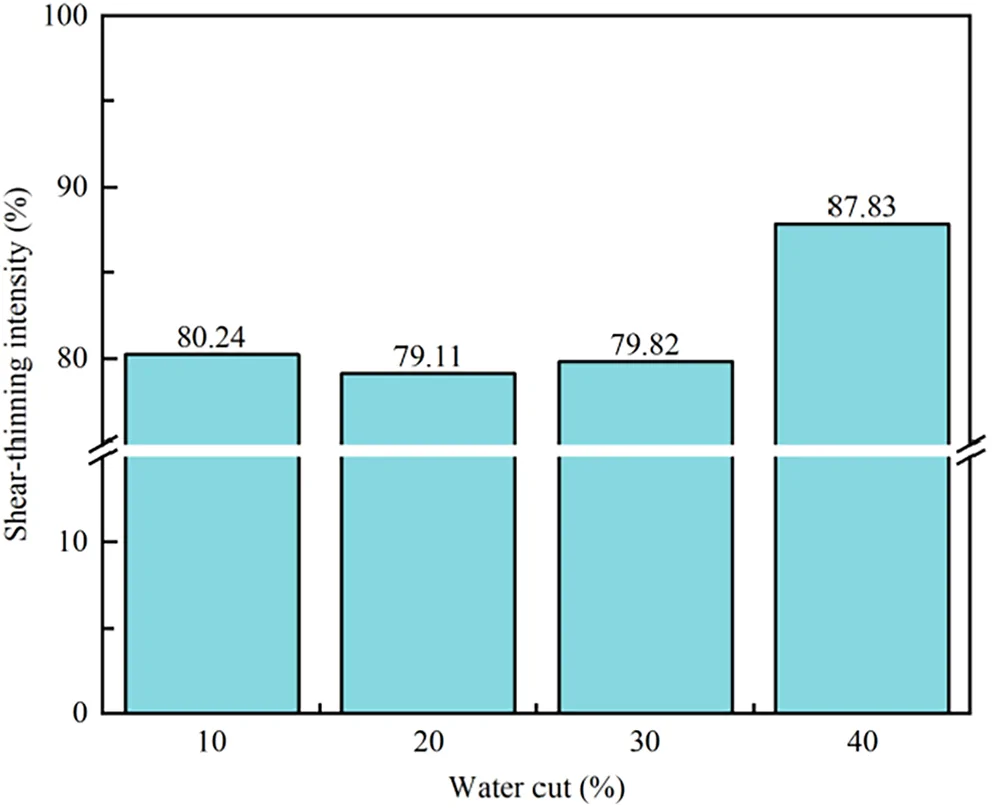
Bar chart of shear-thinning intensity *I*_*st*_ *(w)* at various water cuts (at 16 °C).

As illustrated by the shear-thinning intensities under various water cuts in Figure 14, distinct variations in structural response are observed across the systems. Specifically, the 40% water cut system exhibits the highest shear-thinning intensity (87.83%), followed by the 10% (80.24%) and 30% (79.82%) systems, while the 20% system shows the minimum value (79.11%). Taking the in-situ microscopic video of the 40% water cut emulsion at 32 °C under a shear rate of 2 s^–1^ as a representative case, the presence of wax crystal aggregates and wax-droplet co-aggregates is evident. These mesoscopic structural units manifest three distinct kinematic behaviors under shear: (i) integral translation along the flow field while maintaining structural integrity (yellow region); (ii) structural disruption and subsequent reorganization induced by shear forces (red region); and (iii) relative stasis within the fixed field of view (black region). Based on these observations, three distinct structural categories are defined: Structure A refers to units with no observable displacement within the fixed field of view; Structure B comprises units that undergo collective motion with the flow field without significant morphological alteration; and Structure C represents units that experience structural breakdown or reconfiguration, as depicted in Figure 12. It must be emphasized that this classification is a descriptive categorization of motion states based on trackable units within the fixed observation window. Therefore, Structures A, B, and C may consist of wax aggregates, droplets, or complex wax-droplet clusters, with their classification dictated by kinematic features rather than chemical composition. Units migrating out of the field of view during the observation period are excluded from the statistical analysis. The relative proportions of Structures A, B, and C for the 10% and 40% water cut emulsions at 32 °C, under shear rates of 0.5 s^–1^ and 2 s^–1^, were quantified and are summarized in Figure 15 (with black, yellow, and red representing Structures A, B, and C, respectively).

**Figure 15:**
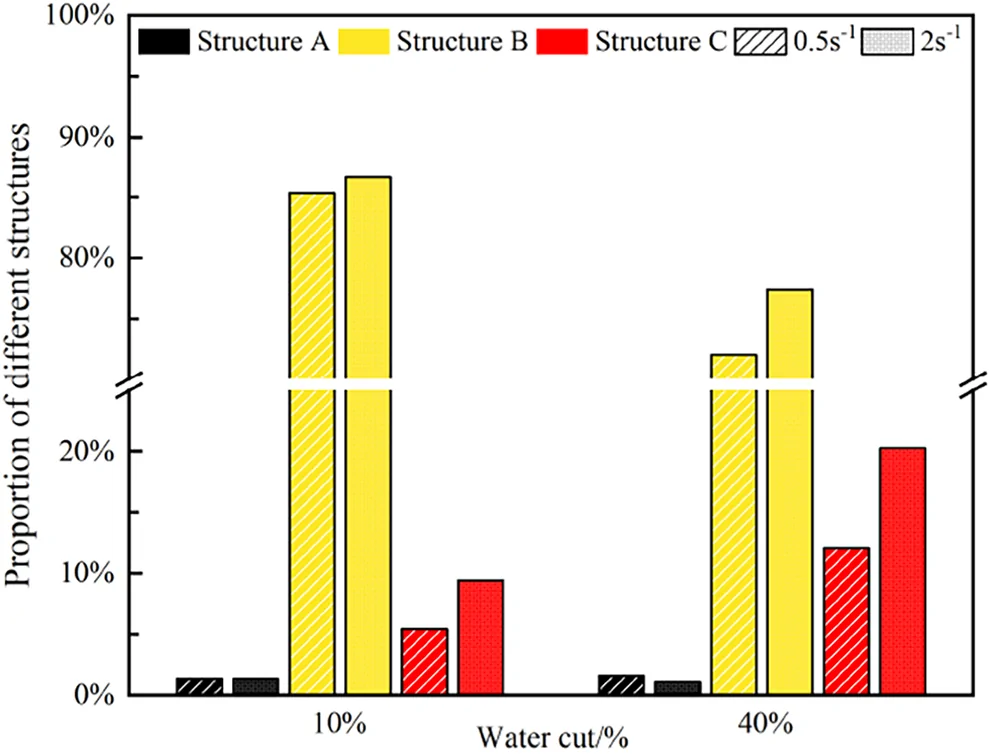
Changes in motion forms of various mesoscopic structures in emulsions with different water cuts under the influence of shear.

As shown in Figure 15, for the 10% water cut emulsion system, as the shear rate increases, the proportion of Structure A decreases from 1.37% to 1.33%, while the proportions of Structure B and C increase from 85.31% and 5.44% to 86.61% and 9.43%, respectively. In contrast, in the 40% water cut emulsion system, after the shear rate increases, the proportion of Structure A drops from 1.59% to 1.10%, and the proportions of Structure B and Structure C rise from 72.05% and 12.05% to 77.41% and 20.28%, respectively; the magnitude of these changes is much larger than that of the 10% water cut system. Combining the previous analysis of mesoscale structures, it is known that when water cut is higher, the mesostructures such as droplet-wax aggregates and wax-droplet-wax co-aggregates are more complex, with enhanced aggregation behavior, leading to increased structural strength. Under shear, these structures with certain strength are destroyed on a large scale, and complex aggregates are broken into many dispersed monomer wax crystals or droplets, which can move fully in the flow field, significantly reducing macroscopic viscosity. Compared with the 10% emulsion, the changes in mesostructure after shear are more significant in the high water cut system, which is directly reflected in a more obvious downward trend of macroscopic viscosity, showing stronger shear-thinning. It should be pointed out that the above microstructure analysis mainly reveals the mechanism of the influence of shear on the viscosity change trend of different water cut systems, while the difference in absolute viscosity values between different water cut systems is determined by the influence of water cut itself on emulsion viscosity, as detailed in Section 3.1.

In summary, the results indicate that the water cut not only dictates the initial structural state of the system but also modulates its capacity for structural disruption and reconfiguration within a shear flow field. From the perspective of mesostructural evolution, the shear-induced viscosity variations are governed by the synergistic regulation of two key factors:

(1) The population distribution of mesostructural units, specifically the relative proportions of Structures A, B, and C, which determine the magnitude of collective motion and structural breakdown.
(2) The intrinsic mechanical properties of these units, namely their resistance to shear deformation and failure, which dictate the susceptibility of the structural network to disruption and reorganization under equivalent shear conditions.

These two factors are closely coupled, collectively determining the macroscopic shear-thinning intensity. Variations in water cut lead to distinct mesoscopic compositions, characterized by differences in the Structures and prevalence of interfacial linkages. Structural units dominated by homo-interfacial contacts (e.g., wax-wax) exhibit high cohesive strength, rendering them resilient to shear-induced disruption. Conversely, composite structural units primarily linked through hetero-interfacial interactions (e.g., wax-droplet) possess lower interfacial strength, making them more prone to slippage, dissociation, and rearrangement. Consequently, the water cut modulates the system’s overall shear response by altering the hierarchy of interfacial linkages, ultimately resulting in the observed differences in shear-thinning intensity across various water cut systems. To address the intrinsic mechanical discrepancies from a microscopic perspective,.i.e., why structural connections dominated by interfacial adsorption are more prone to deformation and failure under shear than the rigid connections formed by direct wax-wax contact, molecular dynamics (MD) simulations will be employed in the following section to analyze the interaction energies of different types of contact interfaces (e.g., wax-wax, wax-water, and multiphase bridging interfaces).

### 3.3 Microscopic Mechanism Analysis Based on Molecular Dynamics Simulation

#### 3.3.1 Simulation Conditions and Methods

Based on the established molecular cluster models of waxy crude oil emulsions, molecular dynamics (MD) simulations were performed to investigate the inter-unit interactions under cooling and shear conditions, aiming to elucidate the mesostructural evolution mechanisms driven by variations in water cut. Integrating the compositional characteristics of the crude oil with the mesoscale microscopic characterization at various water cuts, three representative Structures of interfacial contacts were defined:

(1) Homo-aggregate interfaces, involving contacts between identical species (e.g., wax-wax and droplet–droplet);
(2) Hetero-aggregate interfaces, characterized by cross-phase interactions between dissimilar species (e.g., wax-droplet);
(3) Heterogeneous flocs (hereafter referred to as hetero-flocs) interfaces, featuring multiphasic synergistic configurations (e.g., droplet-wax-droplet).

The fundamental molecular systems comprise n-octadecane (C_18_H_38_), n-octacosane (C_28_H_58_), asphaltenes, resins, and water molecules. All simulations were conducted in the NVT ensemble to ensure consistent thermodynamic conditions for the comparative analysis of interfacial behavior.

To systematically characterize the formation of interfacial structures and their mechanical stability under hydrodynamic disturbances, a two-stage tandem simulation protocol—comprising “spontaneous adsorption” followed by “steered pulling”—was developed, covering 26 distinct cases (Table 3). Phase I (Cases 1–13) represents the aggregation and nucleation stage. The system was linearly cooled from 313.15 to 299.15 K, followed by a 100 ns isothermal relaxation. During this phase, no external forces were applied, allowing intermolecular van der Waals forces and electrostatic interactions to drive the spontaneous structural aggregation typically observed during cooling. Phase II (Cases 14–26) focuses on tensile rupture, employing steered molecular dynamics (SMD) simulations to investigate interfacial stability. Starting from the equilibrated aggregate configurations, core clusters were isolated using a 0.35 nm contact cutoff radius. A constant pulling velocity of 0.001 nm·ps^–1^ was applied to induce interfacial separation, thereby simulating the disruptive effects of a macroscopic shear flow field on the aggregated structures. A core sub-system extraction strategy was employed in this stage to minimize interference from the thermal motion of solvent molecules, enabling a precise quantitative analysis of the interfacial interaction energies and force–displacement characteristics. Specifically, the target molecular cluster was defined as the largest assembly in direct physical contact within a cutoff radius of 0.35 nm. This specific value was selected because it approximates the sum of the van der Waals radii for common non-hydrogen atoms (e.g., C, N, and O, typically 0.34–0.37 nm). As such, it serves as a representative spatial threshold for substantial non-covalent interactions between non-bonded atoms. This choice aligns with established computational standards, ensuring the retention of authentic interfacial contacts while effectively filtering out transient solvent molecules resulting from thermal fluctuations. Consequently, the extracted tensile systems strictly represent physically significant aggregated interfaces.

**Table 3:** Simulation Conditions

Case	Molecular Contact Model	Pulling Velocity (nm·ps^–1^)	Temperature (K)	Interaction Mode	Interfacial Category
1	Wax-Wax	-	313.15 → 299.15	Aggregative Adhesion	Homo-aggregate interfaces
2	Wax-Wax	-	313.15 → 299.15	Aggregative Adhesion	
3	Wax-Wax	-	313.15 → 299.15	Aggregative Adhesion	
4	Wax-Wax	-	313.15 → 299.15	Aggregative Adhesion	
5	Wax-Wax	-	313.15 → 299.15	Aggregative Adhesion	
6	Droplet–Droplet	-	313.15 → 299.15	Aggregative Adhesion	
7	Wax-Droplet	-	313.15 → 299.15	Aggregative Adhesion	Hetero-aggregate interfaces
8	Wax-Droplet	-	313.15 → 299.15	Aggregative Adhesion	
9	Wax-Droplet	-	313.15 → 299.15	Aggregative Adhesion	
10	Droplet-Wax-Droplet	-	313.15 → 299.15	Aggregative Adhesion	Hetero-flocs interfaces
11	Droplet-Wax-Droplet	-	313.15 → 299.15	Aggregative Adhesion	
12	Wax-Droplet-Wax	-	313.15 → 299.15	Aggregative Adhesion	
13	Wax-Droplet-Wax	-	313.15 → 299.15	Aggregative Adhesion	
14	Wax-Wax	0.001	299.15	Tensile Rupture	Homo-aggregate interfaces
15	Wax-Wax	0.001	299.15	Tensile Rupture	
16	Wax-Wax	0.001	299.15	Tensile Rupture	
17	Wax-Wax	0.001	299.15	Tensile Rupture	
18	Wax-Wax	0.001	299.15	Tensile Rupture	
19	Droplet–Droplet	0.001	299.15	Tensile Rupture	
20	Wax-Droplet	0.001	299.15	Tensile Rupture	Hetero-flocs interfaces
21	Wax-Droplet	0.001	299.15	Tensile Rupture	
22	Wax-Droplet	0.001	299.15	Tensile Rupture	
23	Droplet-Wax-Droplet	0.001	299.15	Tensile Rupture	Hetero-flocs interfaces
24	Droplet-Wax-Droplet	0.001	299.15	Tensile Rupture	
25	Wax-Droplet-Wax	0.001	299.15	Tensile Rupture	
26	Wax-Droplet-Wax	0.001	299.15	Tensile Rupture	

#### 3.3.2 Aggregation and Rupture Dynamics at Distinct Interfacial Contacts

Representative systems for each interfacial contact category were selected to extract and analyze the simulated characteristic parameters. The corresponding evolutionary profiles were then plotted as follows:

As illustrated in Figure 16, the interfacial interaction energies for all three contact categories exhibit a consistent staged evolution during the 100 ns relaxation process. During the initial phase (0–20 ns), the interaction energies undergo a sharp decline from their initial values. The steep slope of the curves signifies the rapid establishment of attractive intermolecular interactions as pre-assembled wax crystal clusters spontaneously adsorb onto surrounding components during the cooling process, marking a rapid aggregation stage. In the subsequent phase (20–100 ns), the fluctuations in the energy profiles diminish, with the interaction energies converging toward a stable negative plateau. This transition indicates that the interfacial molecular configurations have essentially solidified, and the system has reached a state of aggregation equilibrium. This dual-stage evolution pattern—characterized by a “rapid decline followed by plateau relaxation”—is consistently observed across homo-aggregates and hetero-aggregate interfaces in Figure 16a and b and complex hetero-flocs interfaces in Figure 16c, proving to be independent of specific molecular compositions or contact configurations.

**Figure 16:**
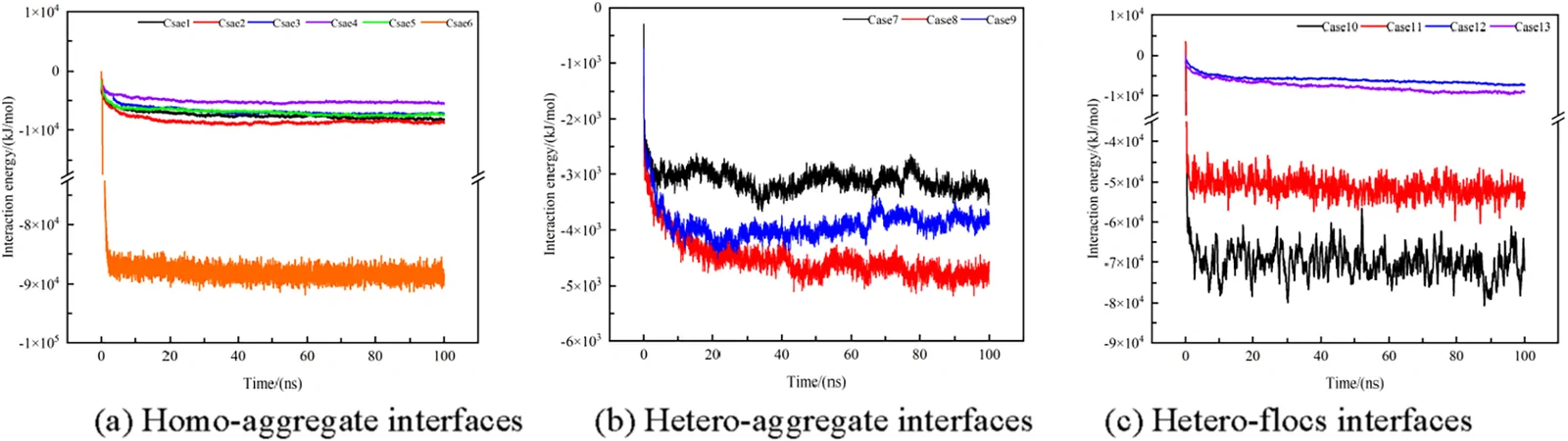
Evolution of interfacial interaction energies for various contact categories during aggregation simulations.

As illustrated in Figure 17, under a constant pulling velocity of 0.001 nm/ps, the interaction energies across the three interfacial systems exhibit a consistent evolutionary trend characterized by a “continuous increase toward zero.” As tensile displacement increases, the interaction energy of each system originates from a negative value at the aggregated equilibrium state and rises monotonically, reflecting the progressive attenuation of intermolecular attractive interactions during forced interfacial separation. In the terminal stage of stretching, the curves converge to approximately zero, signifying the final fracture state in which interfacial molecules achieve complete decohesion. This evolutionary pattern is consistently observed across all three investigated interfaces (Figure 18).

**Figure 17:**
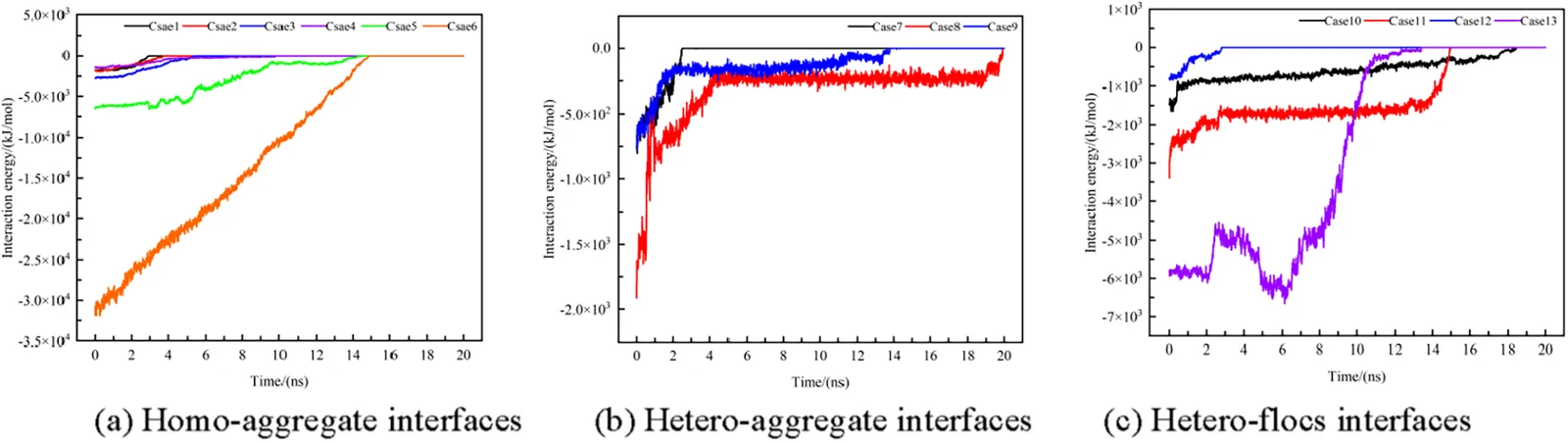
Evolution of interaction energies at various contact interfaces during tensile failure simulations.

**Figure 18:**
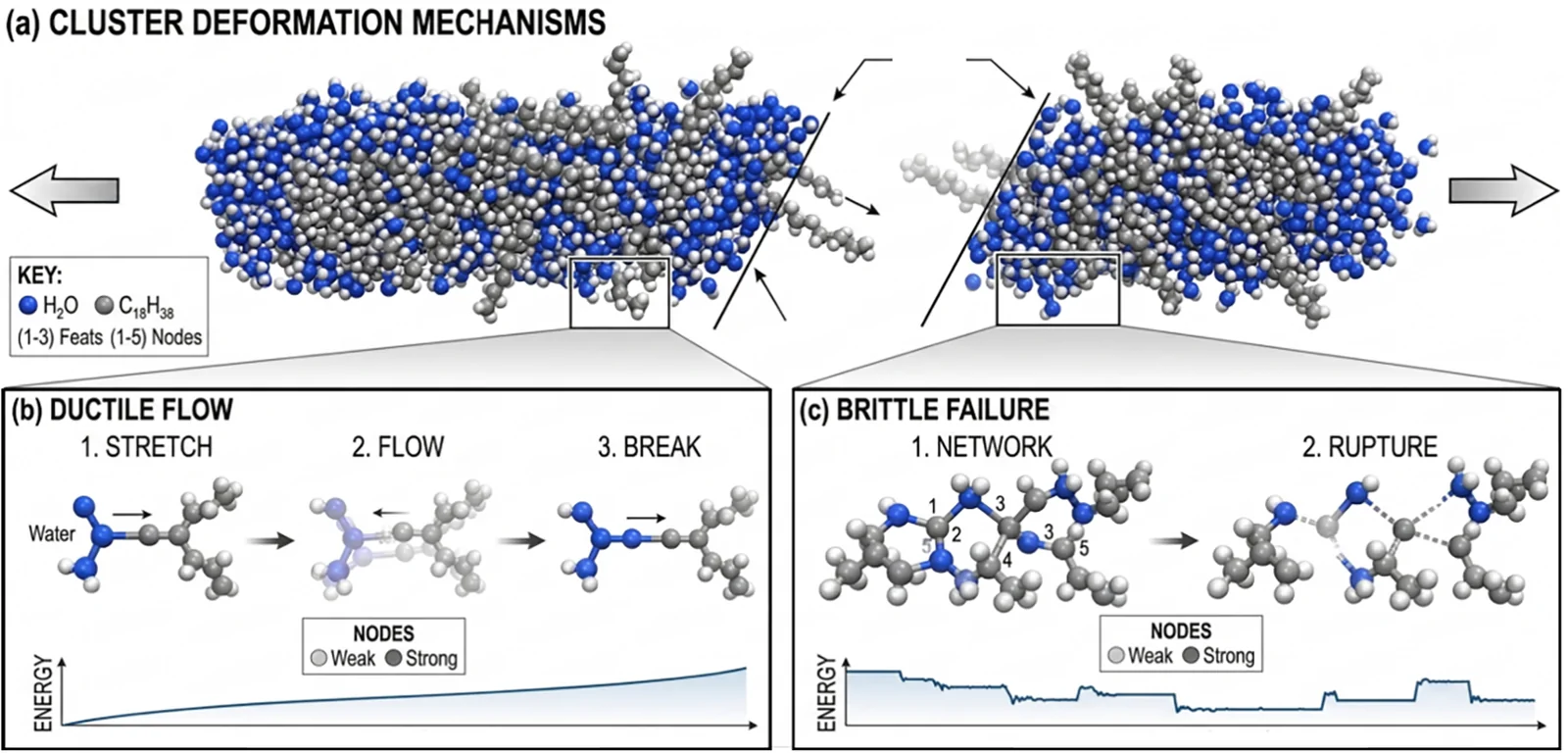
Molecular snapshots of interfacial fracture dynamics.

Distinct variations in fracture dynamics are observed across different interfacial categories. For homo-aggregates in Figure[Fig fig17]a, the interaction energy curves exhibit a smooth, monotonic increase with uniform energy growth, indicating a continuous failure process characterized by a holistic, cooperative decoupling under external loading. Within the hetero-aggregates in Figure 17b, Case 21 (red curve) exhibits a transient energy dip during the initial tensile stage (0–3 ns), followed by a rapid rebound. This phenomenon reveals that the configuration undergoes localized molecular rearrangement at the onset of interfacial stress; subsequently, the interaction energy gradually converges toward zero as displacement proceeds, indicating complete interfacial decoupling upon sufficient separation. The hetero-flocs interfaces in Figure 17c, particularly Case 26 (purple curve), demonstrate the most sophisticated multi-step features; several energy minima are observed within the 5–10 ns interval, followed by stepwise recoveries. This behavior reflects the sequential disintegration of the multiphase bridging network, where weak-binding and strong-binding nodes fracture chronologically, manifesting a characteristic progressive failure mechanism.

As illustrated in Figure 19, the tensile force–displacement profiles across the three interfacial categories exhibit a characteristic fracture evolution: a rapid initial ascent to a peak followed by a fluctuating decline toward zero. During the nascent stage of tension, the system undergoes deformation to counteract the external load, resulting in a sharp escalation of tensile force until the maximum value is reached. This peak signifies the ultimate tensile resistance (i.e., yield strength) of the interface, serving as a direct measure of interfacial adhesion strength. With further displacement, the non-bonded intermolecular interactions are progressively overcome, triggering a fluctuating decay in tensile force. This downward trend persists until the interfaces achieve complete separation, at which point the tensile force converges to approximately zero.

**Figure 19:**
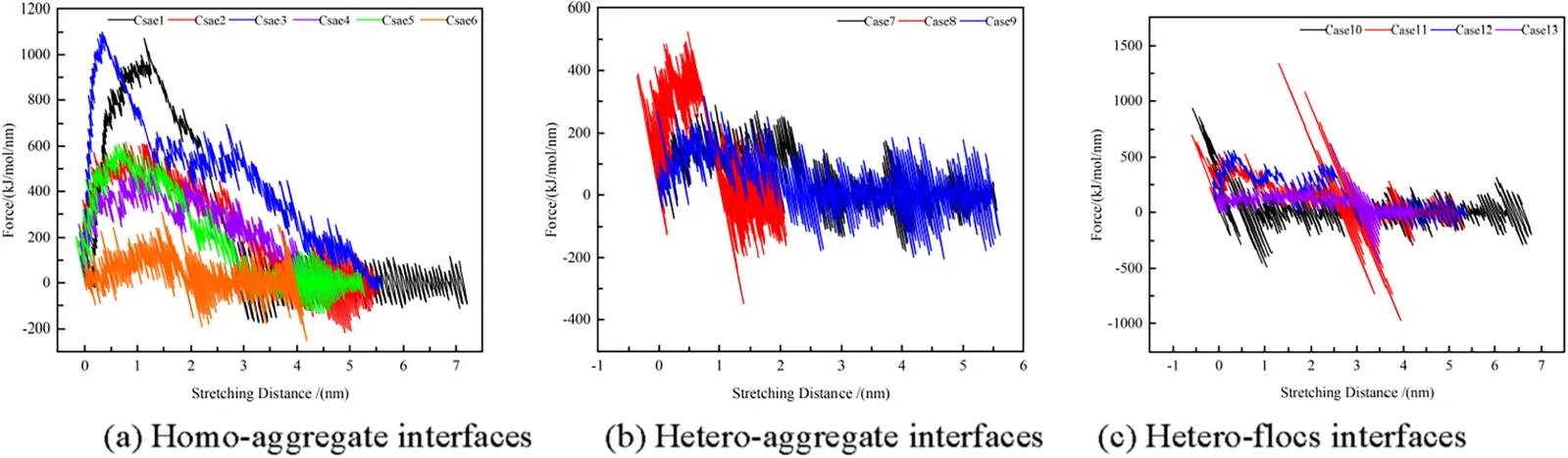
Force–displacement profiles of distinct contact interfaces under steered MD pulling.

A detailed comparison of the dynamic mechanical responses across the three systems reveals distinct interfacial behaviors. Homo-aggregate interfaces in Figure 19a exhibit not only generally higher peak tensile forces (reaching up to approximately 1100 kJ·mol^–1^·nm^–1^) but also extended fracture elongation distances. This observation indicates that the adhesion between homogeneous interfaces is exceptionally robust, conferring the strongest overall resistance to external mechanical disruption. In contrast, hetero-aggregate interfaces in Figure 19b display lower peak tensile forces (predominantly ranging between 200 and 500 kJ·mol^–1^·nm^–1^) coupled with a rapid attenuation of tensile force. This phenomenon reflects relatively weaker interactions across heterogeneous interfaces, rendering them susceptible to interfacial slippage and delamination. Furthermore, the tensile profiles of hetero-flocs interfaces in Figure 19c are characterized by pronounced oscillatory and multi-peak features. The recurring stress fluctuations observed during the descending phase in certain cases further corroborate the sequential disintegration and asynchronous fracture mechanism of the internal multiphase bridging network under applied loading. Consequently, these macroscopic tensile mechanical discrepancies are consistent with the interaction energy evolution trends observed in Figure 17, providing mutual validation.

#### 3.3.3 Comparative Binding Strength of Distinct Contact Interfaces

Although the preceding profile analysis elucidates the dynamic evolution patterns of the three interfacial categories, direct comparisons based solely on absolute interaction energies are inherently limited, as they fail to account for variations in molecular count and contact area across simulation cases. Consequently, to eliminate discrepancies arising from system size effects, the interaction energy per unit area is introduced as a normalized evaluation metric. The effective contact area is quantitatively determined using the solvent-accessible surface area (SASA) - based contact area calculation, a robust approach widely adopted in studies of protein-ligand interactions and colloidal interfaces.^30^ The specific calculation is formulated as follows

2 $$A_{contact}=\frac{{\mathrm{S̅}}_{A}+{\mathrm{S̅}}_{B}-{\mathrm{S̅}}_{total}}{2}$$

where: *S̅*_*A*_ and *S̅*_*B*_ represent the average surface areas of the two independent clusters prior to contact (calculated from trajectory data obtained before and after the tensile simulations), respectively; *S̅*_total_ denotes the total surface area of the clusters in the aggregated state.

Building upon this foundation, the interaction energy per unit area (also termed energy density) is defined as follows

3 $$E_{density}=\frac{E_{\mathrm{int}er}}{A_{contact}}$$

where: *E*_inter_ denotes the total interaction energy between the left and right pulling groups within the system.

Through this normalization procedure, diverse interfacial systems with varying spatial scales and molecular compositions are unified under a consistent comparative baseline, thereby enabling a rigorous quantitative evaluation of interfacial adhesion strength. The calculated normalized interaction energies for all 13 investigated systems are summarized in Table 4.

**Table 4:** Calculated Normalized Interaction Energies for the Various Investigated Systems

Interfacial Category	Effective contact area (nm^2^)	Total interaction energy (kJ·mol^–1^)	Interaction energy per unit area (kJ·mol^–1^·nm^–2^)
Homo-aggregate interfaces	36.01	–1761.58	–48.92
	38.30	–1777.17	–46.40
	47.24	–2766.57	–58.57
	26.81	–1398.41	–52.15
	41.20	–6417.75	–155.77
	5.10	–30758.01	–6031.57
Hetero-aggregate interfaces	31.44	–676.65	–21.52
	82.88	–1913.95	–23.09
	30.65	–772.18	–25.19
Hetero-flocs interfaces	83.62	–1477.44	–17.67
	165.80	–3393.56	–20.47
	49.69	–828.99	–16.68
	315.48	–5819.47	–18.45

Overall, the interaction energies per unit area across the three interfacial categories exhibit distinct variations. Specifically, the homo-aggregate interfaces (excluding Case 6) yield a mean value of approximately −72.36 kJ·mol^–1^·nm^–2^, signifying exceptionally robust and stable interfacial adhesion. In contrast, the hetero-aggregate interfaces and hetero-flocs interfaces exhibit lower magnitudes of approximately −23.27 kJ·mol^–1^·nm^–2^ and −18.32 kJ·mol^–1^·nm^–2^, respectively, with the latter demonstrating the weakest interactions among the three groups. Consequently, an explicit hierarchy of interfacial adhesion strength is established: homo-aggregate interfaces > hetero-aggregate interfaces > hetero-flocs interfaces.

It is noteworthy that the interaction energy per unit area for Case 6 reaches approximately −6031.57 kJ·mol^–1^·nm^–2^, significantly exceeding those of the other scenarios within the same category. This exceptionally high value is attributed to the superposition of strong intermolecular hydrogen bonding and electrostatic interactions at the droplet–droplet interface.^31^ In the in situ microscopic observations, mesoscopic configurations of water droplets encapsulated by asphaltene/resin interfacial films and forming “droplet–droplet” bridging structures were clearly captured (e.g., Figure 9, 10% water, 40 °C). The molecular model in Case 6 represents the interfacial contact between two water droplets stably coated by asphaltene and resin molecules, and is therefore consistent with this experimentally observed configuration. It does not correspond to direct, unprotected water–water contact, which is rare in real emulsions without film rupture. Topologically, Case 6 is classified under “homo-aggregate” interfaces because it involves the contact between two identical-phase entities (water droplets), consistent with the classification criterion adopted throughout this study. However, its dominant interaction mechanism (hydrogen bonding and electrostatic forces between asphaltene/resin-coated interfaces) is fundamentally distinct from that governing the remaining homo-aggregate cases (van der Waals forces between wax crystals). To avoid conflating these mechanistically different scenarios, Case 6 is excluded from the statistical mean of the homo-aggregate category, which is intended to reflect the representative interfacial strength of wax crystal–wax crystal contacts in the emulsion network. The individual value for Case 6 is nevertheless reported to provide a mechanistic reference for the asphaltene/resin-bridged droplet–droplet contact.

The underlying physical mechanisms governing the aforementioned hierarchy can be elucidated at the molecular scale. Homo-aggregate interfaces (predominantly wax crystal-wax crystal) are composed of identical molecules with similar chemical properties. These interfaces exhibit high steric compatibility among molecules and a high density of van der Waals contacts between alkane chains, thereby yielding the highest binding energy per unit area. In contrast, hetero-aggregate interfaces (e.g., wax crystal-emulsified water droplet) involve cross-phase interactions between polar and non-polar components. The disparities in chemical properties reduce the structural compatibility of molecular arrangements at the interface, which consequently decreases the effective contact density and attenuates the interfacial binding energy. For hetero-flocs interfaces (characterized by multiphase bridging configurations), steric hindrance effects and geometric mismatches between distinct phases impede the formation of compact contacts. Instead, the interfacial adhesion is dominated by weak point- or line-contacts, culminating in the lowest interaction energy per unit area. These quantitative findings are consistent with the dynamic failure characteristics observed in the tensile profiles, providing cross-validation from an energetic perspective. Specifically, the smooth, continuous fracture behavior of homo-aggregate interfaces corresponds to their maximum binding energy per unit area, whereas the progressive, multi-step failure of hetero-flocs interfaces aligns perfectly with their minimum interfacial adhesion strength. Ultimately, these dual analyses—from both energetic and dynamic process perspectives—mutually substantiate the reliability of the proposed interfacial adhesion hierarchy and its underlying physical interpretation.

In addition to the interaction energy, the mechanical responses of the systems during the tensile fracture process are inherently governed by the contact area. To further elucidate the mechanical failure resistance properties of the contact interfaces, the peak stress per unit area (σ_max_) and the work of fracture per unit area (*G*) are introduced as quantitative metrics. These parameters are mathematically formulated as follows

4 $$\sigma_{\max}=\frac{F_{\max}}{A_{contact}}$$

5 $$G=\frac{W}{A_{contact}}=\frac{\int \mathrm{Fdx}}{A_{contact}}$$

where: σ_max_ denotes the ultimate capacity of the interface to resist fracture, possessing dimensions equivalent to those of pressure; *G* characterizes the energy absorption capacity of the interface throughout the fracture process.The calculated normalized peak stresses and tensile works for the various investigated systems are summarized in Table 5.

**Table 5:** Calculated Normalized Peak Stresses and Works of Fracture for the Various Investigated Systems

Interfacial Category	Effective contact area (nm^2^)	Peak stress (kJ·mol^–1^·nm^–1^)	Work of fracture (kJ·mol^–1^)	Peak stress per unit area (kJ·mol^–1^·nm^–3^)	Work of fracture per unit area(kJ·mol^–1^·nm^–2^)
Homo-aggregate interfaces	36.01	1072.45	1776.30	29.78	49.33
	38.30	611.89	1381.09	15.98	36.06
	47.24	1099.35	2510.23	23.27	53.14
	26.81	529.33	1176.55	19.74	43.88
	41.20	1617.74	1076.33	39.26	26.12
	5.10	308.94	145.30	60.58	28.49
Hetero-aggregate interfaces	31.44	286.77	166.79	9.12	5.31
	82.88	524.24	293.06	6.33	3.54
	30.65	317.98	148.89	10.37	4.86
Hetero-flocs interfaces	83.62	932.47	130.35	11.15	1.56
	165.80	1339.41	688.16	8.08	4.15
	49.69	573.93	562.58	11.55	11.32
	315.48	624.39	392.64	1.98	1.24

Overall, the tensile mechanical properties across the three interfacial categories exhibit a distinct hierarchical disparity. The homo-aggregate interfaces (excluding Case 6) demonstrate robust and stable interfacial adhesion; their mean peak stress per unit area and mean work of fracture per unit area reach high values of 25.61 kJ·mol^–1^·nm^–3^ and 41.71 kJ·mol^–1^·nm^–2^, respectively. In contrast, the mechanical performance of hetero-type interfacial systems undergoes severe degradation. For hetero-aggregate interfaces, these two respective metrics decrease substantially to 8.61 kJ·mol^–1^·nm^–3^ and 4.57 kJ·mol^–1^·nm^–2^. Similarly, the hetero-flocs interfaces manifest comparable mechanical fragility, yielding a mean peak stress of a mere 8.19 kJ·mol^–1^·nm^–3^ and a mean work of fracture of approximately 4.57 kJ·mol^–1^·nm^–2^.

As evidenced by Table 5, the normalized mechanical parameters further corroborate the previously established hierarchy of interfacial adhesion strength. The homo-aggregate interfaces not only exhibit superior peak stresses per unit area (averaging >20 kJ·mol^–1^·nm^–3^) but also yield works of fracture per unit area that are significantly higher than those of the other configurations. This indicates that the compact molecular packing among homologous molecules confers substantial deformation resistance and facilitates massive energy dissipation during the fracture process. In stark contrast, both σ_max_ and *G* for the hetero-aggregate interfaces and hetero-flocs interfaces experience drastic reductions. The hetero-flocs interfaces, in particular, display pronounced mechanical vulnerability, with their works of fracture per unit area remaining predominantly at low levels (approaching 1.2 kJ·mol^–1^·nm^–2^ in certain cases). This implies that the complex, multiphase bridging networks are highly susceptible to slippage and peeling failures under applied stress, thereby requiring minimal thermodynamic work to achieve complete interfacial separation. Consequently, these quantitative mechanical findings are in excellent agreement with the trends derived from the interaction energy densities, collectively elucidating the stability variations among the distinct microscopic interfaces from the dual perspectives of energetics and mechanics.

#### 3.3.4 Cross-Scale Correlation Model between Mesoscopic Structures and Microscopic Interfaces

The macroscopic rheological behavior of waxy crude oil emulsions under shear inherently reflects the concurrent evolution of microscopic intermolecular interactions and mesoscopic structural configurations. Given the inherent limitations of conventional optical microscopy in directly visualizing the dynamic arrangement of nanoscale interfacial molecules, a semi-quantitative multi-scale mapping model is proposed by coupling mesoscopic topological descriptors with microscopic thermodynamic and mechanical parameters.

This model assumes that the local resistance of the system to shear-induced structural breakdown is predominantly governed by microscopic interfacial interactions. To construct a rigorous validation framework, both thermodynamic and mechanical metrics are incorporated: the interfacial energy density (*E*_density_) characterizes the thermodynamic attributes of the interface, while the maximum stress per unit area (σ_max_, interfacial strength) and the fracture energy per unit area (*G*, interfacial toughness) serve as the principal mechanical characteristic parameters.

Prior to establishing the mapping relationships, the proportional distributions of microscopic contact interfaces within the mesoscopic structural units (Structures A, B, and C) are first quantitatively defined. Based on mesoscopic statistical observations, the numerical ratios of Structure A, B, and C units vary with water cut. Accordingly, representative values at characteristic water cut conditions are selected to define the proportional distributions of mesoscopic - microscopic contact types, as summarized in Table 6.

**Table 6:** Proportional Distributions of Mesoscopic-Microscopic Contact Structures

Mesoscopic dynamic structural units	Proportion of homo-aggregates	Proportion of hetero-aggregates	Proportion of hetero-flocs
Structure A	75%	20%	5%
Structure B	15%	75%	10%
Structure C	10%	20%	70%

As outlined in Table 6, the proportional distributions of Structure A, B, and C units are derived from mesoscopic statistical observations. Because the numerical ratios of Structures A, B, and C vary with water cut, representative values obtained under characteristic operating conditions are reported in Table 6. The proportional distributions of contact interfaces were determined through systematic image analysis across multiple fields of view obtained during synchronous in-situ rheo-microscopic observations.^54,55^ For each structural unit category (Structures A, B, and C), at least 20 representative micrographs were examined across different water-cut conditions. The contact interface types (homo-aggregate, hetero-aggregate, and hetero-flocs) within each unit were manually identified based on their morphological characteristics: homo-aggregate interfaces were identified by the presence of continuous, same-phase contact domains (e.g., wax crystal networks or clustered water droplets); hetero-aggregate interfaces were characterized by distinct cross-phase boundaries (e.g., wax crystals adsorbed onto water droplet surfaces); and hetero-flocs interfaces were recognized by the presence of multiphase bridging configurations (e.g., droplet–wax–droplet chains). The reported percentages in Table 6 represent averaged values from all analyzed fields of view, with an uncertainty of approximately ±5%. The underlying physical rationales are as follows: Structure A represent continuous network structures that remain relatively stationary within the flow field and exhibit robust shear resistance. The internal nodes of such dense phases (e.g., continuous wax crystal networks or clustered water droplets) are predominantly surrounded by like-kind molecules. Consequently, these units are dominated by like-aggregate interfaces with high binding energies, while unlike-aggregate contact interfaces appear only sparingly at the structural periphery. Structure B manifest as stable dispersed phases that translate with the flow field without undergoing rupture. This dynamic stability implies that the internal cohesive energy of these units exceeds the energy input from local shear deformation. Morphologically, this configuration resembles a “core-shell structure” stabilized by interfacially adsorbed surface-active components (e.g., wax-coated water droplets stably dispersed in the continuous phase). Because the energetic focus of the system is concentrated at the phase boundaries, these units are predominantly composed of hetero-aggregate interfaces with intermediate interaction energies. Structure C are highly susceptible to yielding and fracture under applied shear, and their disintegration directly leads to a macroscopic decrease in the apparent viscosity and flow resistance of the continuous phase. Within complex multiphase bridging networks (e.g., water droplet-wax crystal-water droplet configurations), steric hindrance and molecular packing misalignment substantially weaken interfacial adhesion, resulting in an abundance of weak point—or line-contact configurations. Therefore, Structure C are predominantly composed of hetero-flocs interfaces exhibiting the lowest binding energies.

Building upon the aforementioned multi-parameter physical framework, a correlation mapping matrix between the mesoscopic structural units and the microscopic interfaces is formulated. For each category of mesoscopic structural unit (denoted as the *k*-th class, where *k* ∈ {*A*,*B*,*C*}), the equivalent interfacial binding energy density (*E̅*_*k*_), equivalent interfacial strength (σ̅_max.*k*_), and equivalent interfacial fracture toughness (*G̅*_*k*_) are explicitly defined as follows

6 $${\mathrm{E̅}}_{k}=\sum_{i}\omega_{\mathrm{ki}}\cdot E_{density,i}$$

7 $${\mathrm{\sigma ̅}}_{\max,k}=\sum_{i}\omega_{\mathrm{ki}}\cdot \sigma_{\max,i}$$

8 $${\mathrm{G̅}}_{k}=\sum_{i}\omega_{\mathrm{ki}}\cdot G_{i}$$

where: ω_*ki*_ denotes the proportion of the *i*-th contact interface within the *k*-th structural unit; *E*_density,*i*_, σ_max,*i*_, and *G*_*i*_ represent the mean attribute values of the corresponding microscopic interfaces, respectively.

By integrating the proportional distributions provided in Table 6 with the mean values derived from the preceding molecular dynamics simulations, the equivalent attributes for each structural unit are calculated as follows

(1) Structure A:
$${\mathrm{E̅}}_{A}=0.75\times \left(-72.36\right)+0.20\times \left(-23.27\right)+0.05\times \left(-18.32\right)=-59.84\mathrm{kJ}\cdot {\mathrm{mol}}^{-1}\cdot {\mathrm{nm}}^{-2}$$
$$\sigma_{\max,A}=0.75\times \left(31.44\right)+0.20\times \left(8.61\right)+0.05\times \left(8.19\right)=25.71\mathrm{kJ}\cdot {\mathrm{mol}}^{-1}\cdot {\mathrm{nm}}^{-3}$$
$$G_{A}=0.75\times \left(34.62\right)+0.20\times \left(4.57\right)+0.05\times \left(4.57\right)=27.11\mathrm{kJ}\cdot {\mathrm{mol}}^{-1}\cdot {\mathrm{nm}}^{-2}$$
(2) Structure B:
$${\mathrm{E̅}}_{B}=0.15\times \left(-72.36\right)+0.75\times \left(-23.27\right)+0.10\times \left(-18.32\right)=-30.13\mathrm{kJ}\cdot {\mathrm{mol}}^{-1}\cdot {\mathrm{nm}}^{-2}$$
$$\sigma_{\max,B}=0.15\times \left(31.44\right)+0.75\times \left(8.61\right)+0.10\times \left(8.19\right)=11.99\mathrm{kJ}\cdot {\mathrm{mol}}^{-1}\cdot {\mathrm{nm}}^{-3}$$
$$G_{B}=0.15\times \left(34.62\right)+0.75\times \left(4.57\right)+0.10\times \left(4.57\right)=9.08\mathrm{kJ}\cdot {\mathrm{mol}}^{-1}\cdot {\mathrm{nm}}^{-2}$$
(3) Structure C:
$${\mathrm{E̅}}_{C}=0.10\times \left(-72.36\right)+0.20\times \left(-23.27\right)+0.70\times \left(-18.32\right)=-24.71\mathrm{kJ}\cdot {\mathrm{mol}}^{-1}\cdot {\mathrm{nm}}^{-2}$$
$$\sigma_{\max,C}=0.10\times \left(31.44\right)+0.20\times \left(8.61\right)+0.70\times \left(8.19\right)=10.59\mathrm{kJ}\cdot {\mathrm{mol}}^{-1}\cdot {\mathrm{nm}}^{-3}$$
$$G_{C}=0.10\times \left(34.62\right)+0.20\times \left(4.57\right)+0.70\times \left(4.57\right)=7.58\mathrm{kJ}\cdot {\mathrm{mol}}^{-1}\cdot {\mathrm{nm}}^{-2}$$

Evidently, the structural units exhibit a consistent hierarchical paradigm in terms of both thermodynamic stability and mechanical fracture resistance (Structure A > Structure B > Structure C). To further establish the transmission pathway from the microscopic and mesoscopic scales to macroscopic rheological characteristics, a comprehensive evaluation metric (Φ_sys_) for the overall shear resistance of the system is formulated based on the multi-scale equivalence principle

9 $$\Phi_{sys}=\sum_{k\in \in \left\{A,B,C\right\}}f_{k}\cdot \left(\alpha \frac{\left|E_{k}\right|}{E_{ref}}+\beta \frac{\sigma_{\max,k}}{\sigma_{\mathrm{ref}}}+\gamma \frac{G_{k}}{G_{\mathrm{ref}}}\right)$$

where: *f*_*k*_ denotes the macroscopic proportion of the *k*-th mesoscopic structural unit within the flow field (which evolves dynamically with applied shear rate and time); α,β,γ represents the dimensionless weight coefficients for the thermodynamic and mechanical parameters (satisfying α + β + γ = 1); *E*_ref_, σ_ref_, *G*_ref_, designates the reference baseline values utilized to achieve dimensional normalization.

By *f*_*k*_ capturing the macroscopic flow field-driven topological evolution of the mesoscopic populations, while simultaneously characterizing the intrinsic shear-resistant properties of the distinct interfaces via the microscopic equivalent parameters (enclosed in brackets), this model successfully actualizes a rigorous cross-scale mathematical correlation encompassing “microscopic interfacial mechanisms—mesoscopic structural evolution—macroscopic rheological characteristics.”

### 3.4 Multi-Scale Structural Disruption and Regulation Strategies for Waxy Crude Oil Emulsion Systems

By integrating macroscopic rheological experiments, mesoscopic in-situ microscopic observations, and microscopic molecular dynamics simulations, the shear-induced structural disruption mechanisms of waxy crude oil emulsions can be elucidated from a multi-scale perspective (Figure 20). The applied shear flow field not only alters the macroscopic flow state of the system but also drives the dynamic reconstruction of the mesoscopic topological networks and microscopic molecular packing. In terms of force transmission, the applied shear exhibits a pronounced “top-down” characteristic: the macroscopic shear stress propagates sequentially through the mesoscopic wax crystal-emulsified water droplet flocculation networks and their constituent aggregates, ultimately concentrating at the microscopic contact interfaces within individual mesoscopic structural units. This cross-scale force transmission subsequently initiates a “bottom-up” cascade of structural disruption. At the microscopic scale, the molecular linkages within the hetero-flocs interfaces—i.e., the multiphase bridging configurations with the weakest binding strength—are the first to progressively loosen and ultimately fracture. As shear persists and structural damage accumulates, molecular rearrangement and dissociation extend progressively throughout the system, driving the sequential failure of hetero-aggregate interfaces (wax crystal–water droplet) and, ultimately, homo-aggregate interfaces (wax crystal–wax crystal). This sequential decoupling of microscopic interfaces translates directly to the mesoscopic scale, manifesting as the progressive disintegration of structural units. Specifically, Structure C (dominated by hetero-flocs interfaces), which exhibit the lowest shear resistance, preferentially undergo localized fragmentation and degrade into smaller aggregates. This is followed by the progressive disintegration of Structure B (dominated by hetero-aggregate interfaces), culminating in the complete rupture of the densely packed Structure A (dominated by homo-aggregate interfaces). This stepwise network degradation at the mesoscopic level directly causes a sharp reduction in the macroscopic rheological parameters of the system, such as yield stress and apparent viscosity.

**Figure 20:**
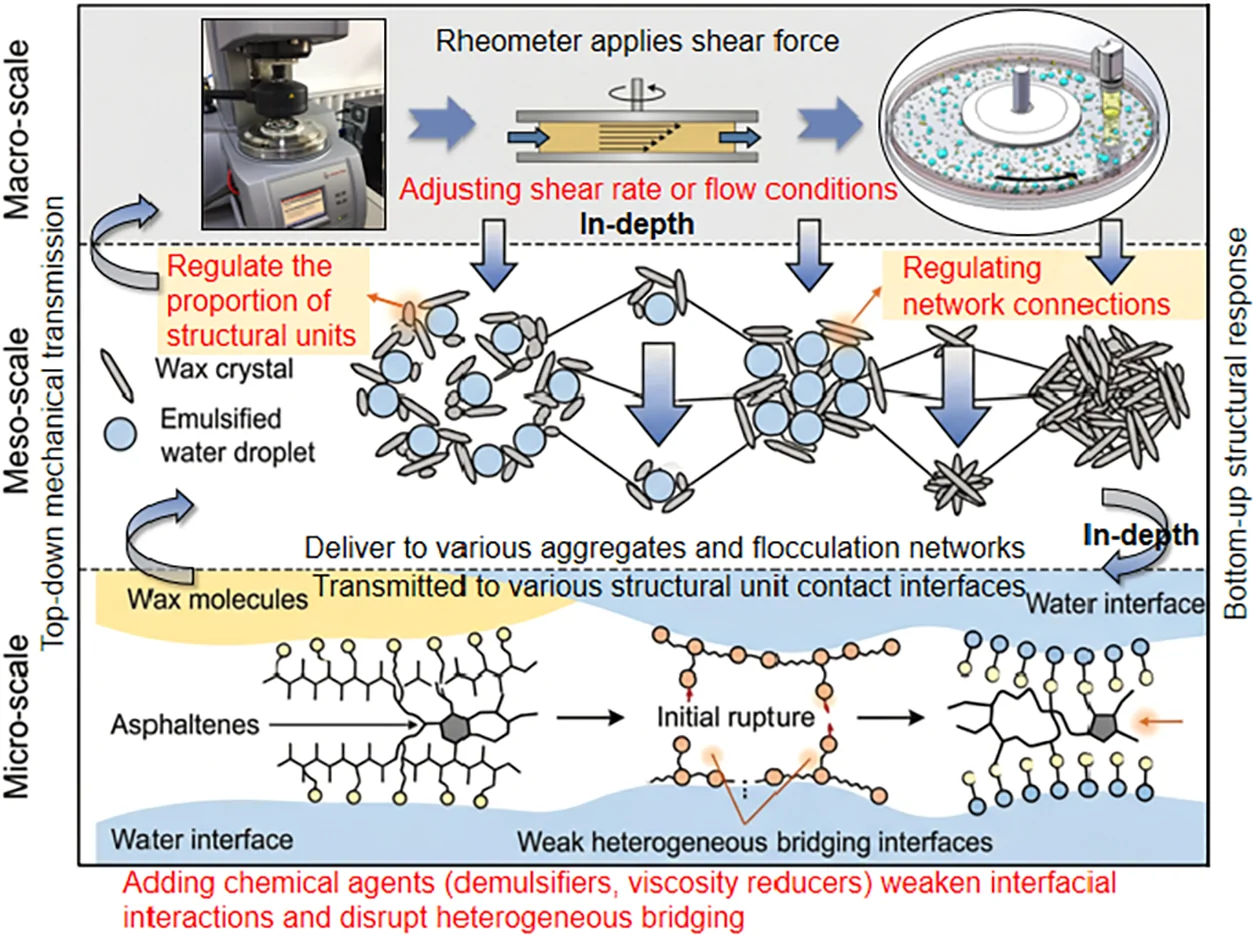
Schematic illustration of the shear-thinning mechanism and corresponding regulation strategies.

The multi-scale structural disruption mechanisms identified above provide a physicochemical basis for the targeted regulation of the macroscopic rheological properties of waxy crude oil emulsion systems. In the research and application of chemical additives (e.g., viscosity reducers, demulsifiers), priority should be given to identifying the “mechanical weak links” within the structural hierarchy as effective targets for intervention. Specifically, at the microscopic level, chemical interventions should selectively attenuate the “multiphase bridging hetero-aggregate interfaces” characterized by low binding energies; at the mesoscopic level, efforts should focus on suppressing the formation and stability of the mechanically vulnerable Structure C. By selectively disrupting or destabilizing these critical weak nodes, a progressive breakdown of the entire network structure can be triggered with minimal energy input, thereby enabling the highly efficient regulation of the macroscopic flow resistance of waxy crude oil emulsions.

It should be acknowledged that the present study possesses several limitations that warrant further investigation. First, the molecular dynamics simulations utilized simplified binary *n*-alkane models (*n*-octadecane, C_18_H_38_, and n-octacosane, C_28_H_58_) to represent the wax fraction. In real crude oils, waxes comprise polydisperse mixtures of *n*-alkanes with broader chain length distributions, iso-alkanes, and cycloalkanes.^56^ The inclusion of more realistic polydisperse wax compositions may modulate interfacial interaction energies and aggregation kinetics, potentially leading to quantitative variations in the calculated mechanical metrics. Second, the cross-scale correlation framework was established using a single waxy crude oil sample from the Hulunbuir oilfield. While the underlying mechanistic principles are expected to be broadly applicable, specific quantitative relationships (e.g., the structural proportions in Table 6) are inherently oil-specific.^33^ Validating this framework across crude oils with varying wax contents, SARA fractions, and wax appearance temperatures remains an essential direction for future research. Third, the present investigation focused on steady-state shear conditions; extending this cross-scale methodology to transient flow scenarios (such as pipeline restart and shear-rate fluctuations) will further enhance its practical engineering relevance.

## 4 Conclusions

Focusing on W/O waxy crude oil emulsions, this study employed a comprehensive multi-scale methodology—integrating simultaneous rheological and in-situ optical microscopic measurements, quantitative mesoscopic structural analyses, and microscopic molecular dynamics simulations—to elucidate the cross-scale regulatory mechanisms governing the rheological behavior of these emulsions. The inherent correlations among macroscopic rheological behavior, mesoscopic structural evolution, and microscopic molecular interfacial interactions form the central theme of this study. The principal conclusions are as follows:

(1) Macroscopic rheological characteristics and water cut effects: As temperature decreases, the apparent viscosity of W/O waxy crude oil emulsions exhibits a staged escalation, accompanied by increased flow resistance. The water cut exerts a pronounced positive amplification effect on macroscopic viscosity: under isothermal conditions, transitioning from low to high water cuts increases the system viscosity by several fold to one order of magnitude. The application of an external shear flow field induces pronounced shear-thinning in the system. Notably, emulsions at higher water cuts exhibit more pronounced shear-thinning, indicating that their internal network structures possess a greater sensitivity to applied shear.
(2) Mesoscopic structural evolution and its regulatory impact on macroscopic rheology: Simultaneous rheological and in-situ microscopic measurements reveal that, during the cooling process, wax crystals and emulsified water droplets cooperatively construct a spatial network structure. Under high water cut conditions, this network becomes markedly denser, resulting in fractal dimensions and droplet number densities significantly higher than those of low water cut systems. The abundance of densely distributed droplets acts as discrete resistance units that impede fluid flow, providing a mesoscopic mechanistic explanation for the substantial viscosity increase associated with elevated water cuts. Furthermore, under applied shear, an increase in shear rate induces a more pronounced increase in the fraction of Structure C in high water cut systems compared to low water cut counterparts. Because these units are dominated by readily rupturable heterogeneous phase contacts, this confirms that the progressive breakdown of the mesoscopic network is the physical origin of macroscopic shear thinning.
(3) Microscopic interfacial mechanisms and their determinative role in mesoscopic structural failure: Molecular dynamics simulations demonstrate fundamental differences in the dynamic failure modes across different contact interfaces: homo-aggregate interfaces undergo smooth, continuous fracture; hetero-aggregate interfaces experience localized rearrangements during the initial stages of loading; and hetero-flocs interfaces exhibit progressive, multi-step failure. Quantitative comparative analyses of the normalized interaction energy per unit area, peak stress, and work of fracture establish a distinct hierarchy in the mechanical failure resistance of the microscopic interfaces. Specifically, the binding energies and fracture resistance of homo-aggregate interfaces are substantially higher than those of hetero-aggregate interfaces, whereas the multiphase-bridged hetero-flocs interfaces exhibit the lowest mechanical resistance. These thermodynamic and mechanical analyses collectively confirm that the heterogeneous, multiphase-bridged interfaces represent the “weak links” within the overall mechanical network, and that this microscopic fragility inherently dictates the preferential failure of mesoscopic Structure C under applied shear.
(4) Macro-meso-micro cross-scale correlations and shear-induced disruption mechanisms: Based on the multi-scale equivalence principle,^32^ the established mapping model quantitatively demonstrates that the equivalent interfacial binding energy density, interfacial strength, and fracture toughness of the mechanically weaker Structure C are substantially lower than those of the stable Structure A, with differences of several fold. Accordingly, a comprehensive cross-scale mechanism for the shear-induced disruption of emulsions is proposed. Macroscopic shear stresses preferentially act upon the multiphase-bridged heterogeneous interfaces, which harbor the weakest microscopic binding forces, subsequently triggering a “bottom - up”, sequential disintegration of the mesoscopic network (Structure C → Structure B → Structure A), and ultimately manifesting macroscopically as a sharp decline in the flow resistance of the system. This mechanism establishes a cross-scale transmission pathway from “microscopic interfacial molecular configurations” through “mesoscopic aggregate structural disruption” to “macroscopic viscosity variation”. Based on these findings, it is recommended that the development of flow assurance chemical additives prioritize the selective weakening of heterogeneous bridging interfaces, as a means of efficiently reducing macroscopic flow resistance.

## Data Availability

The data that has been used is confidential.
